# Vitamin D Supplementation, Serum 25(OH)D Concentrations and Cardiovascular Disease Risk Factors: A Systematic Review and Meta-Analysis

**DOI:** 10.3389/fcvm.2018.00087

**Published:** 2018-07-12

**Authors:** Naghmeh Mirhosseini, Jacqueline Rainsbury, Samantha M. Kimball

**Affiliations:** ^1^Pure North S'Energy Foundation, Calgary, AB, Canada; ^2^Faculty of Nursing, University of Calgary, Calgary, AB, Canada; ^3^St. Mary's University, Calgary, AB, Canada

**Keywords:** vitamin D, cardiovascular, blood pressure, lipids, inflammation, parathyroid hormone, arterial stiffness, meta-analysis

## Abstract

**Background:** Cardiovascular disease (CVD) risk factors are associated with low serum 25 hydroxyvitamin D (25(OH)D) concentrations in observational studies; however, clinical trial findings are inconsistent.

**Objective:** We assessed the effect of vitamin D supplementation and increased serum 25(OH)D concentrations on CVD risk factors in a systemic review and meta-analysis of randomized controlled trials (RCTs).

**Design:** MEDLINE, CINAHL, EMBASE, and Google Scholar were searched for RCTs that evaluated vitamin D supplementation and cardiovascular outcomes [blood pressure, parathyroid hormone (PTH), serum high-sensitivity C-reactive protein (hs-CRP), total cholesterol, high and low density lipoprotein (HDL and LDL, respectively), triglycerides, peak wave velocity (PWV) and Augmentation Index (AI)] from 1992 through 2017. Meta-analysis was based on a random-effects model and inverse variance method to calculate standardized mean difference (SMD) as effect sizes, followed by a leave-one-out method for sensitivity analysis. Risk of publication bias was assessed using Cochrane checklist and Begg funnel plots. The systematic review is registered as CRD42015025346.

**Results:** We identified 2341 studies from which 81 met inclusion criteria. The meta-analysis indicated a significant reduction in systolic blood pressure (SMD = −0.102 ± 0.04 mmHg, 95% confidence interval (CI), −0.20 to −0.03), diastolic blood pressure (SMD = −0.07 ± 0.03 mmHg, 95% CI, −0.14 to −0.006), serum PTH (SMD = −0.66 ± 0.08 ng/L, 95% CI, −0.82 to −0.49), hs-CRP (SMD = −0.20 ± 0.07 mg/L, 95% CI, −0.34 to −0.06), total cholesterol (SMD = −0.15 ± 0.06 mmol/L, 95% CI, −0.25 to −0.04), LDL (SMD = −0.10 ± 0.05 mmol/L, 95% CI, −0.20 to −0.003), triglycerides (SMD = −0.12 ± 0.06 mmol/L, 95% CI, −0.23 to −0.003) and a significant increase in HDL (SMD = 0.09 ± 0.04 mmol/L, 95% CI, 0.00 to 0.17) with vitamin D supplementation. These findings remained significant in sensitivity analyses for blood pressure, lipid profile, serum PTH, and serum hs-CRP. There was no significant effect of vitamin D supplementation on PWV (SMD = −0.20 ± 0.13 m/s, 95% CI, −0.46 to 0.06, *p* = 0.14) and AI (SMD = −0.09 ± 0.14%, 95% CI, −0.37 to 0.19, *p* = 0.52) for vitamin D supplemented groups.

**Conclusion:** These findings suggest that vitamin D supplementation may act to protect against CVD through improving risk factors, including high blood pressure, elevated PTH, dyslipidemia, and inflammation.

## Introduction

The main physiological role of vitamin D has long been regarded as regulation of calcium and phosphorous homeostasis and proper bone mineralization. In more recent years, however, inadequate vitamin D status has been linked to a number of non-skeletal chronic health conditions such as diabetes, cancer, and cardiovascular disease (CVD) (1–3). The prevalence of vitamin D deficiency is high in populations across the globe and an additional 30–50% are at risk of being vitamin D deficient (4, 5). Aging is also associated with decreased vitamin D synthesis in the body, putting individuals already vulnerable at an increased risk of these conditions (6).

Observational studies have consistently found an association between low serum 25-hydroxyvitamin D (25(OH)D) concentrations and presence of CVD risk factors, including blood pressure, dyslipidemia, and inflammation (7–11). A review of prospective studies found that serum 25(OH)D concentrations <25 or 37 nmol/L (10 or 15 ng/mL) were associated with an increased risk of CVD disease or mortality (12). This is supported by a recent meta-analysis that revealed a significant association between low 25(OH)D concentration and increased cardiovascular mortality, a consistent finding across countries, sexes, age groups, and season of blood testing (13).

Current evidence suggests a role for several different vitamins in the protection of proper heart function, especially those with antioxidant potency, and thus multiple vitamin deficiencies may contribute to development of CVD. Antioxidant vitamins such as vitamin C and vitamin E might diminish the rate of oxidative stress which is a crucial component in the pathogenesis of atherosclerosis and CVD. B vitamins, which play a role in ATP energy production, and vitamin D all induce cardioprotective effects and maintain cardiovascular health (14). B vitamins might inhibit homocysteine mediated superoxide production and attenuate the atherogenicity of homocysteine (15), and improve endothelial function through decreasing homocysteine levels leading to increased flow-mediated vasodilation (16). The presence of vitamin D receptor expression in endothelial cells, vascular smooth muscle cells, and cardiac myocytes provides biological support for these observations (17); vitamin D has also been associated with the improvement of endothelial function and glucose homeostasis, reduction of oxidative stress, inflammatory response, and thrombogenesis, as well as the modulation of calcium and lipoprotein metabolism (18). Secondary hyperparathyroidism, excess parathyroid hormone, resulting from chronic vitamin D deficiency has been associated with CVD potentially through several different pathological pathways, including: (1) increased insulin resistance and pancreatic β cell dysfunction, leading to metabolic syndrome and diabetes, (2) activation of renin-angiotensin-aldosterone system (RAAS), increasing blood pressure, leading to apoptosis and fibrosis, and (3) stimulation of systemic and vascular inflammation leading to atherogenesis (4, 19). Current evidence suggests vitamin D deficiency is an important new cardiovascular risk factor that may play a causal role in the development of cardiovascular disease (20).

Several published meta-analyses and systematic reviews have found no beneficial effect of vitamin D supplementation on CVD risk factors (21–26). Ford (24), for example, suggested that there is insufficient evidence to support vitamin D supplementation for the reduction of cardiovascular events, although these authors did raise the possibility that vitamin D supplementation might have an effect on heart failure. Several meta-analyses and systematic reviews have similarly failed to find an association. In their systematic review, Wang et al. (26) showed a statistically nonsignificant reduction in cardiovascular disease with moderate doses of vitamin D (approximately 1,000 IU/d). Mao et al. (25) showed that neither vitamin D supplementation nor calcium supplementation had an effect on major cardiovascular events, myocardial infarction, or stroke. However, a meta-analysis is only as good as the quality of studies included.

The quality of the RCTs included in these meta-analyses has been criticized (27). Many RCTs do not have the ability to detect any effect due to an effect size that is simply too narrow (28). Several RCTs provided vitamin D doses that are far too low to measure a detectable increase in serum 25(OH)D concentration (29–31) and/or are too short in duration (e.g., weeks rather than months or years) to expect a change in health outcomes (32, 33). Further, most of the RCTs were grossly underpowered to detect changes in secondary outcomes (28). Several of the studies do not report baseline and/or follow-up serum 25(OH)D concentrations making it impossible to determine whether a change in vitamin D status occurred and thus whether it can be implicated in observed outcomes.

Given these uncertainties, the question of whether vitamin D supplementation improves cardiovascular risk factors and reduces subsequent disease remains without a convincing answer. The current meta-analysis investigates the role of vitamin D supplementation on cardiovascular outcomes by imposing a stringent set of inclusion criteria for studies by aggregating trials that properly take into account the biology of vitamin supplementation and by understanding the implications of different study designs.

## Methods

### Review design

We conducted a systematic review based on a predefined protocol registered with PROSPERO, International Prospective Register of Systematic Reviews (http://www.crd.york.ac.uk/PROSPERO/display_record.php?ID=CRD42015025346). We included randomized controlled trials that reported blood pressure, total cholesterol, triglyceride, high and low density lipoproteins (HDL and LDL, respectively), as well as parathyroid hormone (PTH) and high sensitivity C-reactive protein (hs-CRP), peak wave velocity (PWV), and augmentation index (AI).

### Search strategy

We searched Medline, the Cochrane Central Register of Controlled Trials, Cumulative Index of Nursing and Allied Health Literature (CINAHL), Excerpta Medica database (EMBASE) and gray literature (i.e., material not published in scientific, peer-reviewed journals) using Google and Google Scholar. We also searched the references of previously published systematic reviews and meta-analyses in this area. The search interval spanned January 1, 1992 through December 31, 2017. Search terms included *vitamin D, vitamin D3, and cholecalciferol* combined with *blood pressure, hypertension, cardiovascular, heart disease, coronary disease, lipids, cholesterol, triglycerides, HDL, LDL, hs-CRP, inflammation, PTH, arterial stiffness, PWV, AI* and *randomized controlled trial*. Studies were limited to those published in English.

### Study selection

#### Inclusion criteria

Only studies that met the following criteria were considered for this systematic review and meta-analysis: (1) studies included participants with any baseline 25(OH)D level; (2) studies recorded changes in blood pressure, PTH, hs-CRP, lipid profile, peak wave velocity, and/or augmentation index; (3) a minimum of 3 months of supplementation/therapy to ensure that the intervention had sufficient time to produce an effect on serum 25(OH)D concentrations; (4) studies with daily, weekly, or monthly frequency of dosage; (5) studies reported pre- and post-serum 25(OH)D levels (or when it was supplied by authors following request); (6) studies with control groups using a placebo and those receiving placebo plus a co-intervention (if both arms of the study received the co-intervention); and (7) studies using vitamin D_3_ or cholecalciferol.

#### Exclusion criteria

Studies were excluded if: (1) they were nonclinical studies, observational studies, case-control, or cross-sectional studies; (2) they were methodological reports, editorials, narrative reviews, comments, and letters; (3) participants were younger than 18 years old; (4) intervention periods were less than 3 months; (5) dosage was less frequent than monthly or if a bolus dose was used; and (6) studies provided inadequate information on outcomes or serum 25(OH)D levels; (7) studies showed on improvement in vitamin D status (serum 25(OH)D change over time ≤ 0).

Two authors (NM, JR) independently reviewed each reference title and abstract to determine whether the studies met the inclusion criteria. Any disagreements with study selection were resolved through the discussion with the third author (SMK). Full-text articles were retrieved for the selected abstracts. Full articles were again assessed by the two independent authors (NM, JR) to ensure that they were eligible to be included in meta-analysis and any disagreements were finalized by the third author (SMK).

### Data extraction

Two independent authors (NM, JR) extracted the following data from the included trials: first author and year of publication; number, age, and sex of participants; study population characteristics; latitude of residence, dosage details of vitamin D including frequency, duration and IU; any co-intervention; pre- and post-serum 25(OH)D levels; pre- and post-measures for blood pressure, PTH, hs-CRP, total cholesterol, LDL, HDL, triglycerides, PWV, and AI. NM or JR also contacted several authors to provide missing data or to clarify data within the primary report. All data was then reviewed by the third author (SMK).

### Risk for bias assessment

We assessed each included study for risk of bias by using fields from the Cochrane checklist (34) to determine the following variables: quality of random allocation concealment, blinding of outcomes assessors, treatment and control group comparability, clear definition of inclusion and exclusion criteria, participant blinding to allocation, selective reporting, if intention-to-treat analysis applied, and description of withdrawals and dropouts. Each criterion was marked as (+) with adequate information, (−) with inadequate information and (?) with unclear information (Table S1).

We generated Begg funnel plots to visually examine possible publication bias. These plots were supplemented by formal statistical testing using the Egger weighted regression tests (35). The analysis for the effects of publication bias was adjusted using the Duval and Tweedie trim-and-fill method (36).

### Strategy for data synthesis and statistical analysis

We performed the meta-analysis at the trial level using Comprehensive Meta-Analysis V3 (Biostat 2014, Englewood, NJ) (37). To calculate the effect size, the mean change in concentrations, calculated as measure at the end of intervention minus measure at baseline, and the standard deviation of the outcomes were used for both treatment groups (38) and the effect size was expressed as standardized mean difference between vitamin D intervention and placebo groups, with a 95% confidence interval. For all treatment effects, a negative value denoted a reduction in the outcomes within the vitamin D group compared with placebo. We used the *I*^2^ index to evaluate heterogeneity among the included studies and with a value ≥50%, random-effects model (using the DerSimonian–Laird and generic inverse variance method) was applied (39, 40).

We conducted a sensitivity analysis using the leave-one-out method to assess the effect of each study on the overall effect size (41). For studies with more than one vitamin D supplemented group (e.g., different daily doses given), the trial with the higher dose was selected and compared with the placebo group.

### Subgroup analysis

To further assess interactions among subgroup treatments and also to address heterogeneity among included studies, we defined *a priori* subgroups as followed: participant's age (<55 vs. ≥55 years old), which was the median of study population's age and the central value of data providing an equal distribution of information for comparison; vitamin D supplementation dose (<4,000 vs. ≥4,000 IU/day), which is the average dose required to provide optimal serum physiological levels of vitamin D (100–130 nmol/L) (30, 42); serum 25(OH)D concentration at the end of the intervention (<86 vs. ≥86 nmol/L), which was selected as the median value of serum 25(OH)D levels; duration of intervention (<6 months vs. ≥6 months), which was selected based on the half-life of serum 25(OH)D and the time required to maintain a steady serum levels and potentially influence other biomarkers (43–45); obesity (BMI < 30 vs. BMI ≥ 30 kg/m^2^) as defined by WHO (46) and based on and the fact that obese individuals need 2–3 times the amount of vitamin D to achieve the same serum 25(OH)D as normal weight individuals (47); vitamin D deficiency at the beginning of the intervention [serum 25(OH)D < 50 vs. ≥50 nmol/L] as defined by IOM (48) and based on evidence demonstrating a strong association between vitamin D deficiency and higher incidence of CVD risk factors (12); and, calcium co-intervention.

## Results

### Study selection

We screened the titles of 2,341 studies after duplicates were removed. After excluding any irrelevant studies, 252 abstracts were retrieved for further examination. Based on the abstracts, 177 full-text articles were assessed to determine whether they satisfied the inclusion criteria. Of these, 86 were excluded from analysis because the intervention was less than 3 months intervention, the frequency of dosage was more than monthly or bolus doses were given, pre- or post-serum 25(OH)D levels were not reported, or the trials were centered on children/ adolescents.

**Figure 1:**
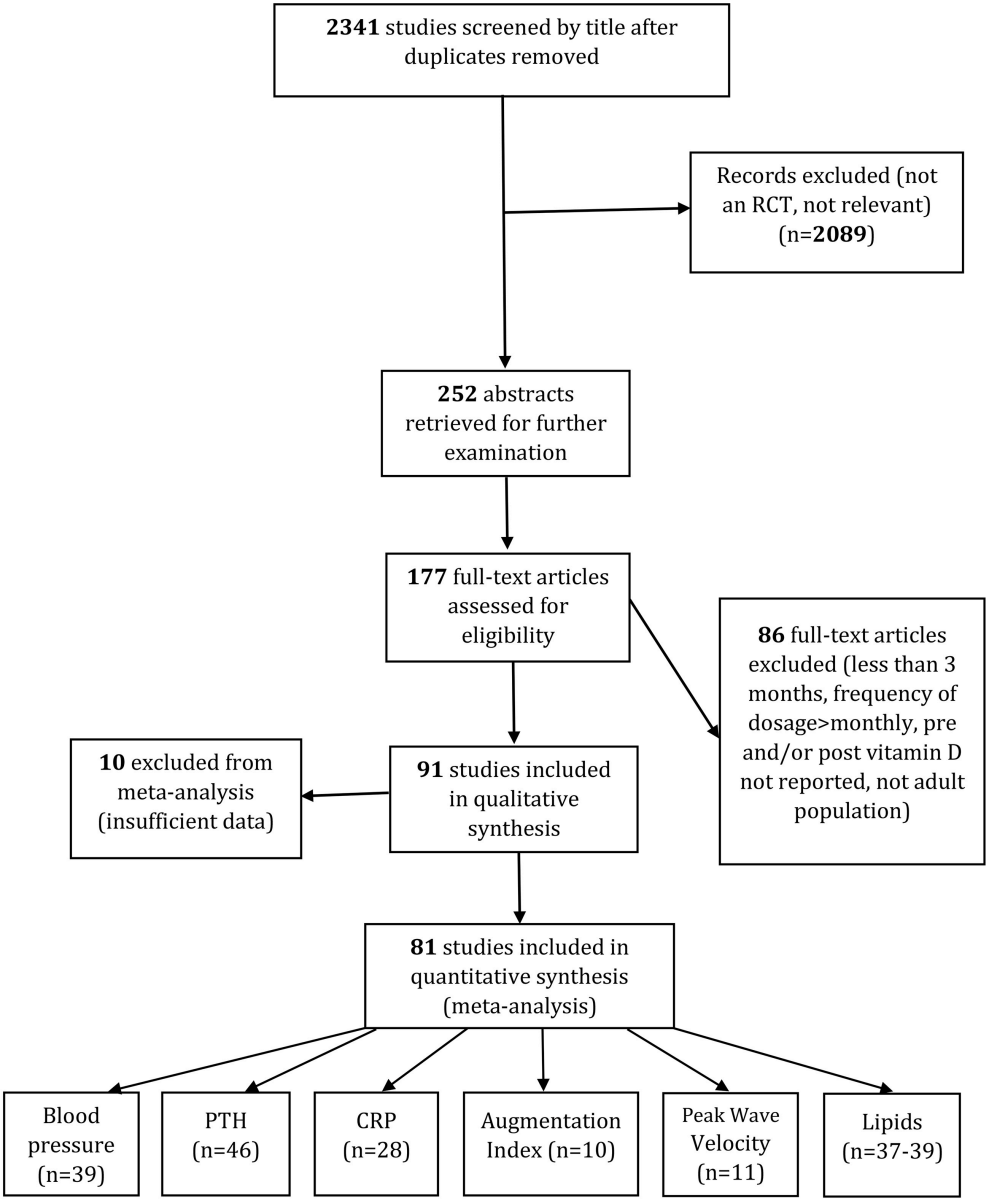
PRISMA diagram of study selection.

For the other 10 trials we did not include data, they either shared similar designs and outcomes (49, 50), had no post serum 25(OH)D data available (51) or, even after contacting the corresponding author, had insufficient information (52–58). We only included papers where an increase in vitamin D status followed supplementation, so the Cooper et al. (59) study was excluded. Finally, 81 studies were included in systematic review and meta-analysis. Details of the complete search process and for each outcome are given in Figure 1.

### Risk of bias assessment

Ten of the included studies lacked information on the blinding of participants and personnel, and one study did not provide information on allocation concealment. Less than half of the studies (*n* = 38) used an intent-to-treat analysis. However, the vast majority of the included studies had a low risk of bias. Details of the quality of bias assessment are provided in Table S1.

### Study characteristics

The characteristics of the included trials are given in Table 1 and studies that were excluded from the meta-analysis are highlighted. The included studies were published between 1992 and 2017. The average latitude of the studies conducted in the northern hemisphere was 43.2 ± 14.7°N (73 studies), with the maximum latitude of 70°N, and the average latitude of the studies from the southern hemisphere was 34.6 ± 6.8°S (eight studies), with the maximum latitude of 43S. Sample sizes varied from 20 (61) to 484 (126); a total of 9,993 participants are included in the meta-analysis, with 5,042 in a vitamin D-supplemented group and 4,951in a placebo group. Twenty of the studies only assessed females (Table 1), but the overall study population included 67% females and 33% males. The mean age of participants ranged from 18 (77) to 85 years (66, 74, 98, 104), with an overall average age of 55 ± 15 years.

**Table 1:** Characteristics of included studies.

	**First author and year**	**N**	**Study population**	**Latitude**	**Mean age**	**% male**	**BMI (kg/m^2^)**	**Daily dose (or daily dose equivalent) of Vitamin D (IU)**	**Other treatment^1^**	**Control group**	**Duration of supplementation**	**CVD outcomes measured**	**Mean baseline 25OHD of treatment group (nmol/l)**	**Mean post treatment 25OHD of treatment group (nmol/l)**	**Mean baseline 25OHD of placebo group (nmol/l)**	**Mean post treatment 25OHD of placebo group (nmol/l)**
1	Alvarez et al. (60)	46	Subjects with early kidney disease	34 N	62	21	NA	7,143 (12 weeks) followed by 3,571 (40 weeks) [4,395 IU/d]		Placebo	12 months	BP, PTH	67	106	80	78
2	Al-Sofiani et al. (61)	20	Type 2 diabetes, insulin resistance & vitamin D deficiency	24 N	48	NA	31	5,000		Placebo	3 months	BP	24	91	39	29
3	Al-Zahrani et al. (62)	183	Type 2 diabetic patients	24 N	55	49	32	6,430 (2 months) followed by 1,500 (1 month) [4,787 IU/d]		Placebo	3 months	BP, Lipid profile	25	83	22	55
4	Arora et al. (63)	534	Individuals 18–50 years old	42 N	37	69	NA	4,000		400 IU Vitamin D/day	6 months	BP	39	83	40	45
5	Barchetta et al. (64)	55	Type 2 diabetes patients with NAFLD	42 N	58	65	30	2,000		Placebo	6 months	Lipid profile CRP BP	43	86	37	40
6	Beilfuss et al. (65)	332	Individuals 21–70 years old overweight & obese	70 N	50	39	34	2,143	500 mg/d Ca	Placebo	12 months	PTH, hs-CRP,	54	99	52	50
7	Bjorkman et al. (66)	119	Long-term bedridden inpatients, age 65+	60 N	85	18	NA	400	daily calcium of 500 mg	Placebo	6 months	PTH, CRP	21	48	24	26
8	Bolton-Smith et al. (67)	123	Healthy, non-osteoporotic women age 60+	56 N	68	0	NA	400	1,000 mg Ca/day (Vitamin D group only)	Placebo	24 months	PTH	63	75	57	49
9	Boxer et al. (68)	64	Patients with heart failure	41 N	66	48	NA	7,143	800 mg Ca/day	800 mg Ca/day	6 months	PTH	48	153	44	45
10	Breslavsky et al. (69)	47	Diabetic patients	32 N	66	47	NA	1,000		Placebo	12 months	BP, lipid profile, CRP, AI	30	44	29	35
11	Bressen dorff et al. (33)	40	Healthy adults, vitamin D deficient	56 N	43	23	25	3,000		Placebo	4 months	BP, PWV, AI	31	88	32	37
12	Cangussu et al. (70)	160	Post-menopausal women	23 S	59	0	NA	1,000		Placebo	9 months	PTH	38	69	42	35
13	Carrillo et al. (71)	23	Overweight & obese adults	40 N	26	48	32	4,000		Placebo	3 months	PTH	52	84	45	59
14	Chandler et al. (72)	149	Healthy Black population 30–80 years old	42 N	51	34	31	4,000	200 mg/d CaCO3	Placebo	3 months	CRP	39	115	38	34
15	Chapuy et al. (73)	142	Ambulatory elderly women living in nursing homes	42–51 N	84	0		800	1,200 mg Ca/day (Vitamin D group only)	Placebo	18 months	PTH	40	105	33	28
16	Chapuy et al. (74)	384	Ambulatory elderly women living in nursing homes	42–51 N	85	0	NA	800	1,200 mg Ca/day (Vitamin D group only)	Placebo	24 months	PTH	23	78	23	18
17	Dalan et al. (52)^*^	64	Type 2 diabetes with hypovitaminosis D	1 N	53	52	28	4,000/2,000		Placebo	4 months	PTH, BP, CRP, AI, Lipid profile	43	79	48	48
18	Cooper et al. (59)^**^	187	Healthy women ≥1 year postmenopausal	34 S	56	0		1,429	1,000 mg Ca/day	Placebo	24 months	PTH	82	81	83	69
19	Daly et al. (75)	124	Healthy, community dwelling men age 50+	38 S	61	100	27	800	1,000 mg Ca/day (Vitamin D group only)	Placebo	24 months	BP, lipid profile, PTH	78	83	76	62
20	Dalbeni et al. (76)	23	Chronic HF patients, vitamin D < 75 nmol/L	45 N	72	74	30	4,000		Placebo	6 months	Lipid profile, PTH, BP	43	79	44	37
21	Dawson-Hughes et al. (72)	389	Healthy, community dwelling, age 65+	42 N	71	45	NA	700	500 mg Ca/day (Vitamin D group only)	Placebo	36 months	PTH	77	112	73	70
22	Dong et al. (77)	44	Normotensive black youth	33 N	18	57	27	2,000		400 IU/d	4 months	PWV, PTH	34	86	33	60
23	Dutta et al. (78)	104	Family member of diabetic patients with IFG/IGT	21 N	55	43	26	8,600 (2 months) followed by 2,000 (10 months) [3,100 IU/d]	CaCO3 125 mg	Placebo	12 months	Lipid, CRP	43	89	45	44
24	El-Hajj et al. (79)	222	Elderly overweight, vitamin D deficient	34 N	71	45	30	3,750	1,000 mg Ca citrate	600 IU/d	12 months	Lipid profile	52	90	50	65
25	Farrokhian et al. (80)	60	Overweight, vitamin D deficient with CAD	34 N	62	50	30	3,571		Placebo	6 months	Lipid profile, CRP	42	86	41	42
26	Forman et al. (81)	142	Healthy Black population	42 N	51	37	31	4,000	200 mg/d Ca	Placebo	3 months	BP	39	115	41	43
27	Forouhi et al. (82)	228	People at risk for type 2 diabetes	52 N	52	57	29	3,333		Placebo	4 months	BP, Lipid profile, CRP, PTH, PWV	46	84	46	39
28	Gagnon et al. (83)	74	Pre-diabetic vitamin D deficient adults	41 S	54	31	31	2,000-6,000 [4,000 IU/d]	1,200 mg CaCO3	Placebo	6 months	Lipid profile, CRP	47	95	43	40
29	Garg et al. (84)	32	Women age 18–35 with PCOS	29 N	22	0	26	4,000	Metformin	Placebo	6 months	Lipid profile, PTH, PWV, AI	19	79	17	17
30	Gepner et al. (85)	98	Post-menopausal women	43 N	63	0	26	2,500		Placebo	4 months	BP, CRP, AI, PWV	76	115	81	80
31	Gepner et al. (86)	110	Post-menopausal women	43 N	61	0	33	2,500		400	6 months	BP, CRP, AI	68	107	63	75
32	Grimnes et al. (87)	94	Healthy adults age 30–75	69 N	53	51	NA	5,714		Placebo	6 months	Lipid profile, PTH	42	143	39	43
33	Harwood et al. (66)^*^	150	Elderly female subjects with recent hip surgery	53 N	81	0		800	1,000 mg Ca/day (Vitamin D group only)	Placebo	12 months	PTH	29	50	30	27
34	Hewitt et al. (88)	60	Vitamin D deficient on hemodialysis	34 S	62	48	NA	4,500		Placebo	6 months	PWV	40	98	48	50
35	Hin et al. (89)	203	Community dwelling elderly	52 N	71	51	28	4,000		Placebo	12 months	PTH	49	137	47	53
36	Holmoy et al. (90)	68	Patients with relapsing remitting MS	60 N	40	29	26	2,857	500 mg Ca/day	Placebo	24 months	PTH	55	123	57	62
37	Islam et al. (91)	75	Healthy women age 16–36	24 N	23	0	22	400		Placebo	12 months	Lipid profile	37	69	35	35
38	Jafari et al. (92, 93)	59	Post-menopausal women with type 2 diabetes	32 N	57	0	29	2,000	Yogurt drink	Placebo (plain yogurt)	3 months	BP, CRP, Lipid profile, PTH	62	87	63	56
39	Jamilian et al. (94)	60	PCOS women	32 N	NA	0	NA	400	Plus Mg 200 mg, zinc 8 mg, calcium 800 mg	Placebo	3 months	Lipid profile	33	62	32	33
40	Jorde and Figenschau (95)	32	Type 2 diabetes	60 N	56	56	32	5,714	Metformin, bed-time insulin	Placebo	6 months	BP, PTH, Lipid profile	60	119	59	57
41	Jorde et al. (49)^#^	438	Overweight or obese subjects	69 N	48	36		5,714 2,857	500 mg Ca/day	Placebo	12 months	BP, lipid profile, PTH	58.7 56.7	140 101	59	57
42	Jorde et al. (50)	227	Prediabetes adults	60 N	62	61	30	2,857		Placebo	5 years	PTH, BP, Lipid profile	60	122	61	67
43	Kamycheva et al. (96)	215	Overweight or obese subjects age 21-70 years	69 N	49	35	35	5,714	500 mg/d Ca	Placebo	12 months	PTH	56	116	53	85
44	Kampmann et al. (54)^*^	15	Adults with type 2 diabetes and hypovitaminosis D	56 N	30	53	33.8	8,400		Placebo	3 months	BP, Lipid profile, PTH, CRP	31	105	35	32
45	Kjaergaard et al. (97)	230	Adults with 25OHD < 55 nmol/l	69 N	64	45	NA	5,714		Placebo	6 months	PTH	47	148	48	53
46	Krieg et al. (98)	72	Women living in nursing homes	47 N	85	0	NA	880	1,000 mg Ca/day (Vitamin D group only)	Placebo	24 months	PTH	30	66	29	14
47	Krul-Poel et al. (99)	261	Adults with type 2 diabetes with no insulin treatment	52 N	67	65	29	1,667	Metformin	Placebo	6 months	BP, PTH	61	101	59	60
48	Larsen et al. (100)	112	Hypertensive patients in Denmark	56 N	60	31	28	3,000		Placebo	5 months	PTH, BP, AI, PWV	58	110	58	50
49	Longenecker et al. (55)^*^	45	HIV-infected vitamin D deficient	41 N	44	75	27.5	4,000	HIV meds	Placebo	3 months	CRP, PTH, BP, Lipid profile	22.5	35	15.5	11
50	Lorvand Amiri et al. (101)	73	Patients with NAFLD, vitamin D deficient	36 N	40	62	30	1,000	Hypocaloric diet (500)	Placebo	3 months	Lipid profile	25	68	25	28
51	Macdonald et al. (102)	179	Healthy post-menopausal women	57 N	65	0	NA	1,000		Placebo	12 months	PTH	33	76	36	32
52	Major et al. (51)^*^		Healthy overweight or obese women	47N	43	0	32	400	1,200 mg Ca/day, calorie restrict diet	Placebo	4 months	BP, Lipid profile				
53	Martins et al. (103)	115	Overweight & obese African American, high BP & vitamin D deficient	34 N	43	61	≥25	3,333		Placebo	3 months	BP, AI, PTH	17	35	17	17
54	Mason et al. (71)	187	Overweight & obese post- menopausal women	47 N	60	0	32	2,000		Placebo	12 months	CRP	54	88	60	50
55	Meyer et al. (104)	65	Nursing home residents	60 N	85	25	NA	400		Placebo	24 months	PTH	47	64	51	46
56	Moreira Lucas et al. (105)	71	Vitamin D deficient & impaired fasting glucose adults	56 N	47	47	31	4,000		Placebo	6 months	BP, PTH, Lipid profile	48	99	48	45
57	Mose et al. (106)	50	Patients on chronic dialysis	57 N	68	64	24	3,000		Placebo	6 months	PTH, CRP, BP, AI, PWV	28	84	28	30
58	Munoz-Aguirre et al. (107)	104	Postmenopausal overweight women with diabetes	18 N	56	0	31	4,000		Placebo	6 months	Lipid profile	55	85	54	56
59	Nikooyeh et al. (108)	60	Diabetic patients	32 N	50	39	29	1,000		Plain yogurt, Ca 300 mg	3 months	BP, Lipid profile	44	78	42	37
60	Patel et al. (109)	24	Type 2 diabetes & vitamin D deficiency	41 N	58	29	32	1,000		400	4 months	PTH, Lipid profile	39	69	44	64
61	Petchey et al. (110)	25	Adult patients with chronic kidney disease	27 S	66	71	NA	2,000		Placebo	6 months	PTH	95	146	88	81
62	Pfeifer et al. (111)	242	Community dwelling, healthy subjects age 70+	46–52 N	77	26	NA	800	1,000 mg Ca/day	Placebo	12 months	PTH	55	84	54	57
63	Pittas et al. (112)	222	Healthy adults	42 N	70	38	26	700	500 mg Ca citrate/day	Placebo	3 years	PTH, CRP	81	111	71	70
64	Qin et al. (113)	56	Statin-treated patients with hypercholesterolemia	40 N	68	55	NA	2,000		Placebo	6 months	Lipid profile, PTH	53	96	53	59
65	Raed et al. (114)	35	Overweight vitamin D deficient African American	40 N	27	18	35	4,000		Placebo	4 months	PWV	33	88	33	34
66	Rahimi-Ardabili et al. (115)	50	PCOS women with vitamin D deficiency	32 N	30	0	29	2,500		Placebo	3 months	PTH, CRP, Lipid profile	17	59	20	21
67	Raja Khan et al. (116)	28	Woman with PCOS	41 N	28	0	37	12,000		Placebo	3 months	CRP, PTH, BP, Lipid profile	50	168	56	56
68	Rajpathak et al. (56)^*^		Post-menopausal women	41N	65	0	29	400	1 g elemental Ca	Placebo	5 years	Lipid profile				
69	Ramly et al. (117)	192	Vitamin D deficient pre-menopausal women	3 N	43	0	NA	7,143 (2 months) followed by 1,667 (10 months) [2,580 IU/d]		Placebo	12 months	BP, lipid profile, PTH	30	86	30	36
70	Rosenblum et al. (118)	71	Overweight & obese adults	42 N	40	18	30	300	Plus 1,050 mg Ca	Placebo	4 months	PTH, Lipid profile	65	77	68	68
71	Ryu et al. (119)	64	Patients type 2 diabetes	38 N	56	NR	NA	2,000	200 mg Ca/day	Placebo	6 months	BP, CRP, lipid profile, PTH	31	86	27	46
72	Sadiya et al. (120)	82	Patients with type 2 diabetes	25 N	49	18	NA	6,000 (3 months) followed by 3,000 (3 months) [4,500 IU/d]		Placebo	6 months	BP, CRP, lipid profile, PTH	29	62	31	25
73	Salekzamani et al. (121)	71	Healthy adults 30-50 years old	38 N	40	49	<40	7,143		Placebo	4 months	BP, lipid profile	16	78	23	21
74	Salehpour et al. (32)	77	Overweight and obese adults	36 N	38	0	30	1,000		Placebo	3 months	BP, PTH, Lipid profile	37	75	47	52
75	Schleithoff et al. (122)	93	Patients with congestive heart failure	51 N	56	83	NA	2,000	500 mg Ca/day	Placebo	9 months	BP, CRP, PTH	36	103	38	47
76	Scragg et al. (123)	304	Healthy adults	43 S	48	25	NA	6,667 (2 months) followed by 3,333 (16 month) [3,700 IU/d]		Placebo	18 months	BP	73	124	71	56
77	Seibert et al. (124)	105	Healthy adults	51 N	45	33	24	800		Placebo	3 months	Lipid, BP	38	72	38	30
78	Shab-Bidar et al. (125)	80	Patients with type 2 diabetes	35 N	52	43	29	1,000	340 mg Ca/d	Placebo	3 months	BP, lipid profile,	39	72	38	33
79	Sinha-Hikim et al. (112)	80	Latino & African American with prediabetes & hypovitaminosis D	34 N	52	30	33	12,185		Placebo	6 months	CRP	55	175	55	55
80	Sollid et al. (126)	484	Subjects with prediabetes	70 N	62	61	NA	2,857		Placebo	12 months	BP, CRP, lipid profile, PTH	60	106	61	65
81	Sun et al. (127)	81	Healthy adults	36 N	43	36	22	420		Placebo	12 months	BP, Lipid profile, PTH, CRP	33	61	32	31
82	von Hurst et al. (57)^*^	235	Women of South Asian origin living in New Zealand	37 S	42	0		4,000		Placebo	6 months	BP, hsCRP, lipid profile	21	80	19	29
83	Tomson et al. (128)	203	Old people living in UK	55 N	71	50	27	4,000		Placebo	6 months	BP, AI, PWV	50	137	50	53
84	Toss et al. (129)	45	Community dwelling subjects	58 N	70	29	NA	1,600	1,000 mg Ca/day	Placebo	12 months	PTH	50	84	47	46
85	Wamberg et al. (130)	43	Obese adults with low Vitamin D levels	55 N	40	29	NA	7,000		Placebo	26 weeks	BP, CRP, lipid profile, PTH	33	110	34	47
86	Witham et al. (131)	50	Patients with chronic fatigue syndrome	56 N	49	24	29	1,667		Placebo	6 months	PWV, AI, BP, PTH, Lipid profile,	44	64	48	44
87	Wood et al. (132)	174	Healthy post-menopausal women	57 N	64	0	NA	1,000		Placebo	12 months	BP, CRP, lipid profile, PTH	32	76	36	32
88	Yeow et al. (133)	26	Women with former gestational diabetes	5 N	36	0	NA	4,000		Placebo	6 months	BP, CRP, lipid profile, PTH	36	92	35	29
89	Yousefi Rad et al. (134)	58	Diabetic patients	32 N	50	NA	28	4,000		Placebo	3 months	Lipid profile	39	69	37	40
90	Yiu et al. (58)^*^	100	Type 2 Diabetes Mellitus patients	22 N	65	50	25	5,000		Placebo	3 months	PTH, PWV, hsCRP, Lipid profile	53	147	55	60
91	Zitterman et al. (135)	165	Healthy overweight subjects	51 N	48	33	NA	3,332		Placebo	12 months	BP, CRP, lipid profile, PTH	30	86	30	42

1 *Given to both groups unless stated otherwise*.

*Highlighted studies were excluded from meta-analysis*,

* *insufficient information*,

** *no improve in serum D*,

# *similar design)*.

Participants received treatment through capsules, pills, tablets, oil drops, or as a specially fortified milk or yogurt drink. Calcium was co-administered with vitamin D and placebo in 24 of the 81 studies (Table 1). The duration of intervention lasted 3 months to 5 years, with an average duration of 9.6 ± 9.2 months (median = 6 months). The daily dose of supplemental vitamin D ranged from 400 (66, 67, 91, 94, 104) to 12,000 IU (116), with an average of 2,967 ± 2,271 IU/day. Baseline serum 25(OH)D concentration varied widely from 16 (121) to 95 nmol/l (110), with the average of 45 ± 16 nmol/L in both vitamin D and placebo groups. The diversity of participants was considerable in these studies. Some were healthy and community dwelling populations, whereas others were institutionalized and/or had specific health conditions such as diabetes, kidney disease, women with polycystic ovary syndrome (PCOS), or included patients on hemodialysis. Forty-six of the studies reported serum PTH concentrations, 39 reported blood pressure and lipid profiles, 28 studies recorded hs-CRP concentrations, and 10/11 studies PWV and AI as their outcomes.

### Effect of vitamin D supplementation on serum 25(OH)D level

Following vitamin D supplementation (average dose of ~3,000 IU/day), there was a significant increase in serum 25(OH)D levels in vitamin D group (48 ± 23 nmol/L) after an average of 9.6 months intervention, while it remained unchanged in placebo group (1 ± 9 nmol/L). Each of the studies reported an overall improvement in vitamin D status, with 27 recording serum 25(OH)D level greater than 100 nmol/L (Table 1). There is a significant dose-response effect between vitamin D supplementation dose and serum 25(OH)D concentration at the end of the intervention (*R*^2^ = 0.37, *p* < 0.001). Achieved serum 25(OH)D concentrations ≥100 nmol/L were observed in trials prescribing vitamin D at doses between 4,000 and 12,000 IU/day (50, 60, 68, 72, 74, 81, 85–87, 89, 90, 95–97, 99, 100, 110, 112, 116, 122, 123, 126, 128, 130, 136, 137).

### Pooled estimate of the effect of vitamin D on cardiometabolic parameters

#### Vitamin D and blood pressure

**Figure 2:**
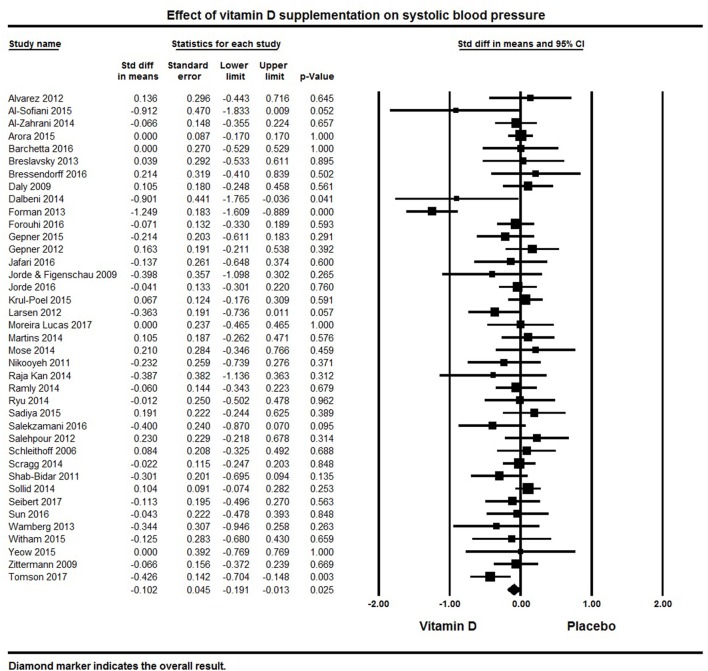
Forest plot detailing standardized mean difference for the impact of vitamin D on systolic blood pressure.

**Figure 3:**
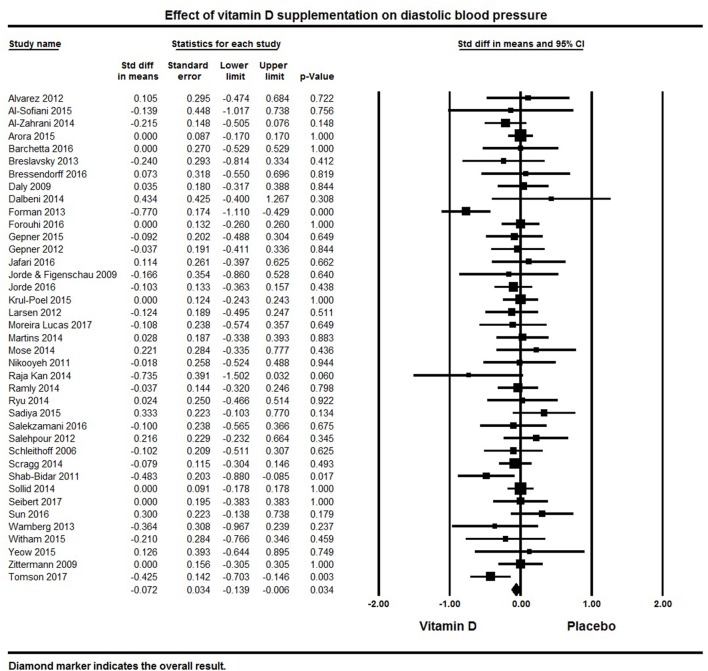
Forest plot detailing standardized mean difference for the impact of vitamin D on diastolic blood pressure.

A total of 39 studies reported on outcomes of systolic and diastolic blood pressure (Figures 2, 3). The pooled effect size (standardized mean difference) of the effect of vitamin D supplementation on systolic blood pressure was −0.102 ± 0.04 mmHg, (95% CI −0.20 to −0.01, *p* = 0.02, *I*^2^ = 51%) across all studies. The pooled effect size for diastolic blood pressure was −0.072 ± 0.03 mmHg (95% CI −0.14 to −0.006, *p* = 0.03, *I*^2^ = 18%) across all studies. Overall results indicated that vitamin D supplementation was significantly associated with lower blood pressure.

Six studies showed a significant reduction in systolic blood pressure (61, 76, 81, 100, 121, 128) and four studies revealed significant reductions in diastolic blood pressure (81, 116, 125, 128). Seventeen studies demonstrated a decreasing trend in systolic and/or diastolic blood pressure following vitamin D supplementation, though these changes were not statistically significant (50, 62, 82, 86, 92, 95, 108, 116, 117, 119, 123–125, 127, 130, 131, 135). The remaining 16 studies with information on systolic blood pressure (32, 33, 60, 63, 64, 69, 75, 85, 99, 103, 105, 106, 120, 122, 126, 133) and 19 on diastolic blood pressure (32, 33, 60, 63, 64, 75, 76, 82, 92, 99, 103, 106, 119, 120, 124, 126, 127, 133, 135) showed either a null effect or an increase in blood pressure. In the majority of these studies, blood pressure was a secondary endpoint and the studies were not designed or powered for detecting the effects of vitamin D supplementation on blood pressure. Some studies also included patients with comorbid condition like kidney (60, 106) or heart failure (122), and others had all or a majority of their participants with normal blood pressure at baseline.

Only one of the included studies centered on hypertensive patients (100). After a five month intervention, this study found a significant reduction in blood pressure following vitamin D supplementation (3,000 IU/day) and improved serum 25(OH)D levels (50 nmol/L increase) compared to placebo.

#### Vitamin D and lipid profiles

**Figure 4:**
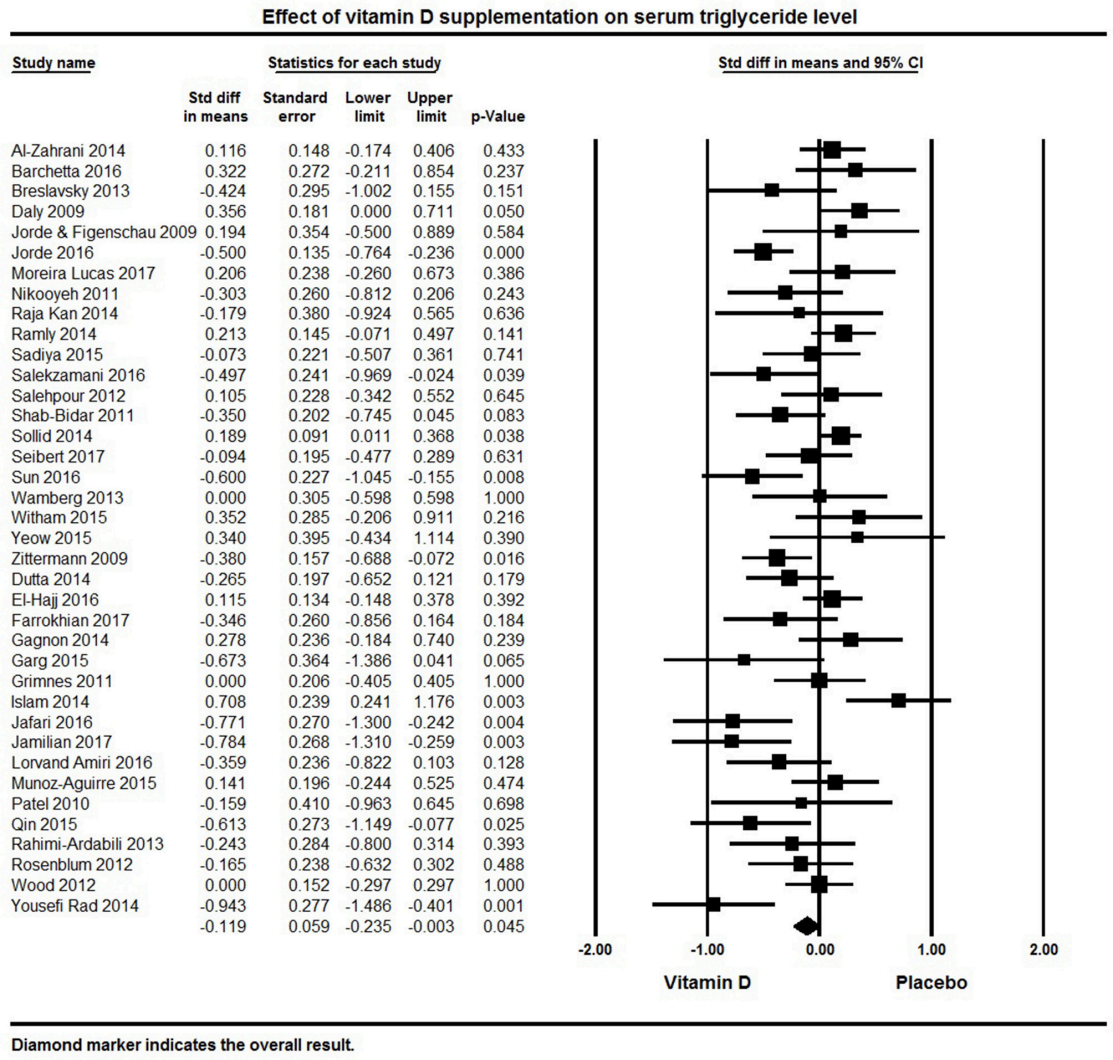
Forest plot detailing standardized mean difference for the impact of vitamin D on serum triglyceride level.

Thirty-eight papers reported on the lipid profiles of participants (Table 1). Across all studies, vitamin D supplementation significantly decreased TG (pooled effect size −0.12 ± 0.06 mmol/L, 95% CI −0.24 to −0.003, *p* = 0.04, *I*^2^ = 64%) (Figure 4). Ten individual studies reported significant reductions in serum triglycerides with vitamin D supplementation (50, 84, 92, 94, 113, 121, 125, 127, 134, 135) and 11 studies indicated a decreasing trend with vitamin D supplementation (69, 78, 80, 101, 108, 109, 115, 116, 118, 120, 124). Seventeen of the 38 studies reported null findings or increased serum TG levels (Figure 4).

**Figure 5:**
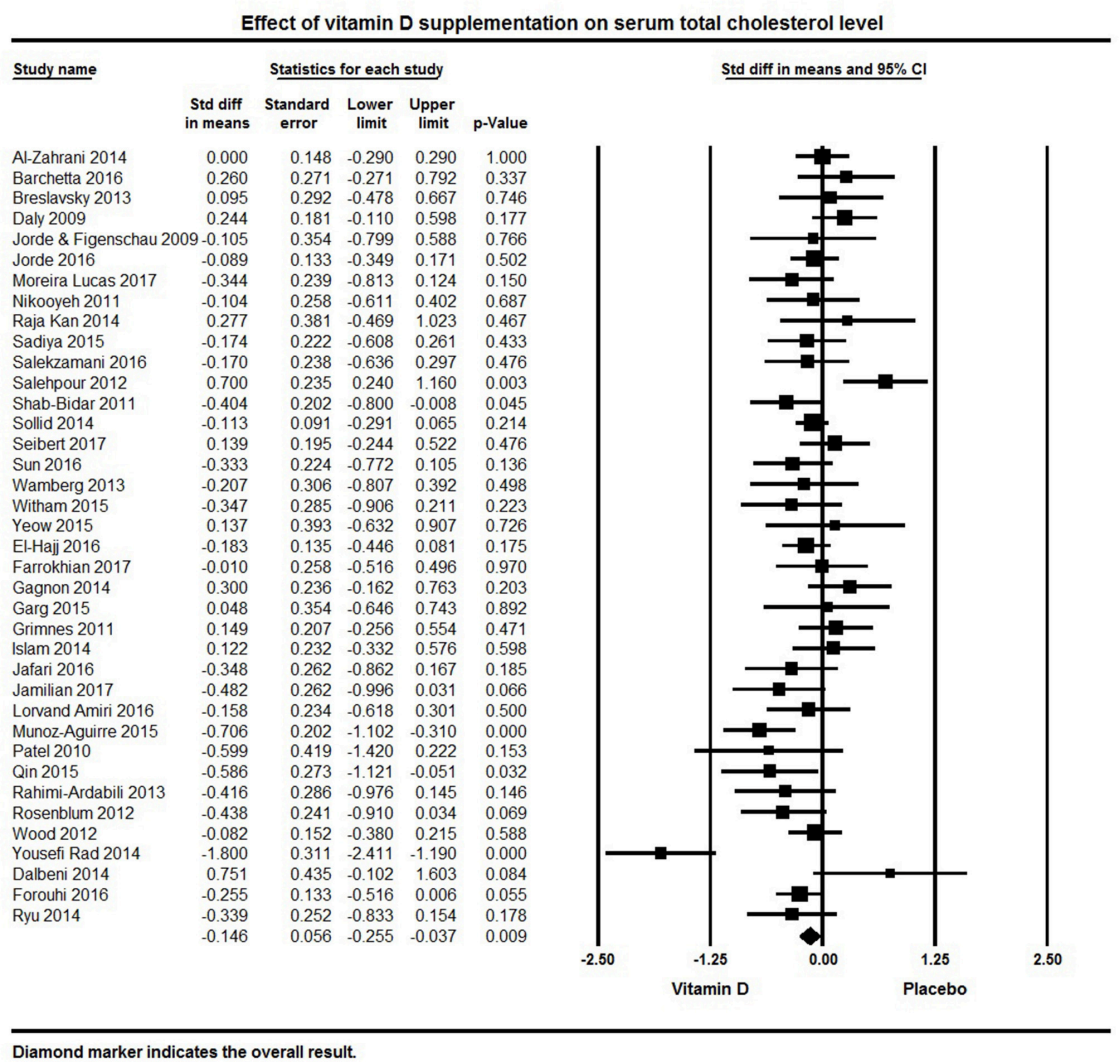
Forest plot detailing standardized mean difference for the impact of vitamin D on serum total cholesterol level.

Thirty-eight studies included in the meta-analysis examined TC levels. The pooled effect size of vitamin D supplementation on TC was −0.15 ± 0.06 mmol/L (95% CI −0.26 to −0.04, *p* = 0.009, *I*^2^ = 57%; Figure 5). Vitamin D supplemented groups had lower TC levels at follow-up in seven individual studies (82, 94, 107, 113, 118, 125, 134), 18 studies found a non-significant trend for lower TC (50, 79, 80, 92, 95, 101, 105, 108, 109, 115, 119–121, 126, 127, 130–132) and 13 studies reported null effect or increased TC levels (32, 62, 64, 69, 75, 76, 83, 84, 87, 91, 116, 124, 133).

**Figure 6:**
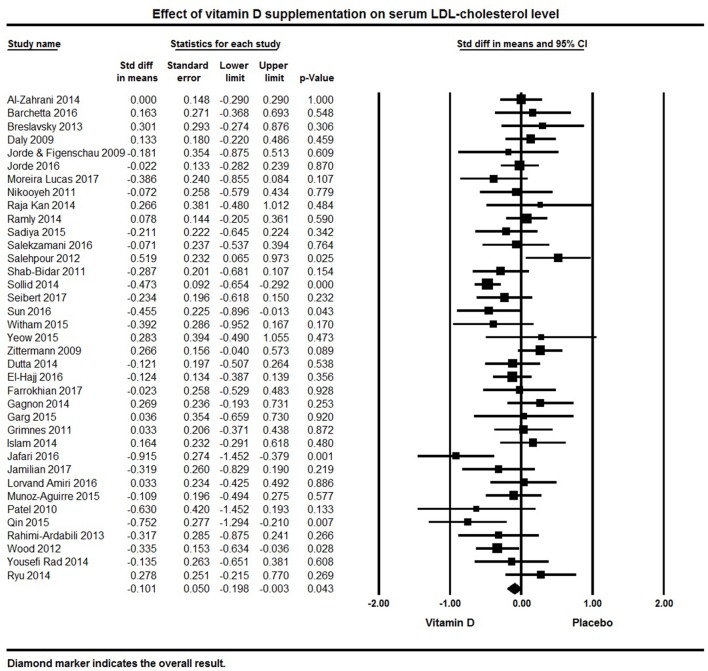
Forest plot detailing standardized mean difference for the impact of vitamin D on serum LDL-cholesterol level.

Thirty-seven studies were included that reported LDL levels. The pooled effect size of vitamin D supplementation on LDL was −0.10 ± 0.05 mmol/L (95% CI −0.20 to −0.003, *p* = 0.04, *I*^2^ = 49%; Figure 6). Vitamin D supplementation was associated with reduced LDL levels in five individual trials (92, 113, 126, 127, 132), 17 studies reported a non-significant trend for decreased serum LDL (50, 78–80, 94, 95, 105, 107–109, 115, 120, 121, 124, 125, 131, 134), and 15 trials did not find any effect on LDL levels (32, 62, 64, 69, 75, 83, 84, 87, 91, 101, 116, 117, 119, 133, 135).

**Figure 7:**
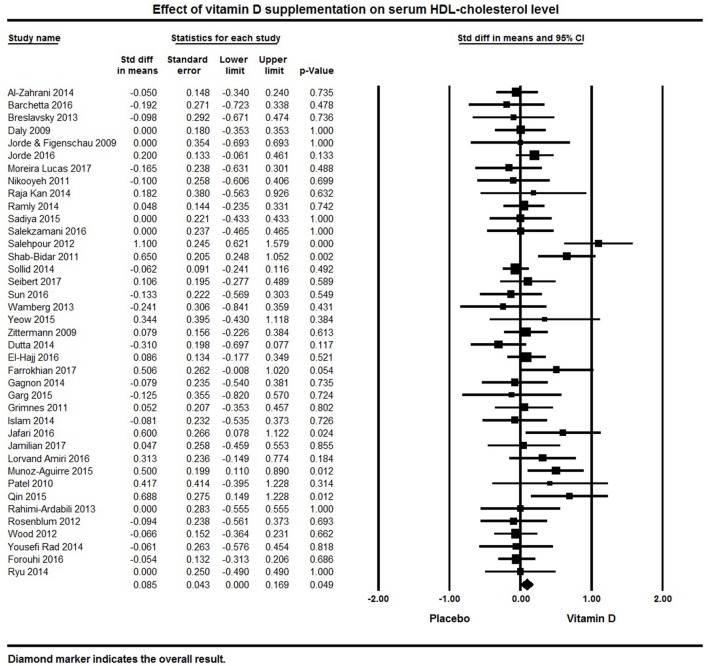
Forest plot detailing standardized mean difference for the impact of vitamin D on serum HDL-cholesterol level.

Serum HDL was a reported outcome for 39 studies. A significant effect of vitamin D supplementation on increased serum HDL was found with a pooled effect size of 0.09 ± 0.04 mmol/L [95% CI 0.00 to 0.17, *p* = 0.05, *I*^2^ = 37%; Figure 7). Vitamin D supplementation significantly increased serum HDL in 6 individual studies (32, 80, 92, 107, 113, 125). Serum HDL cholesterol remained unchanged following vitamin D supplementation in 17 studies (75, 95, 115, 119–121) and an increase in serum HDL levels in 16 studies (62, 64, 69, 78, 82–84, 91, 105, 108, 118, 126, 127, 130, 132, 134) (Figure 7).

#### Vitamin D and PTH

**Figure 8:**
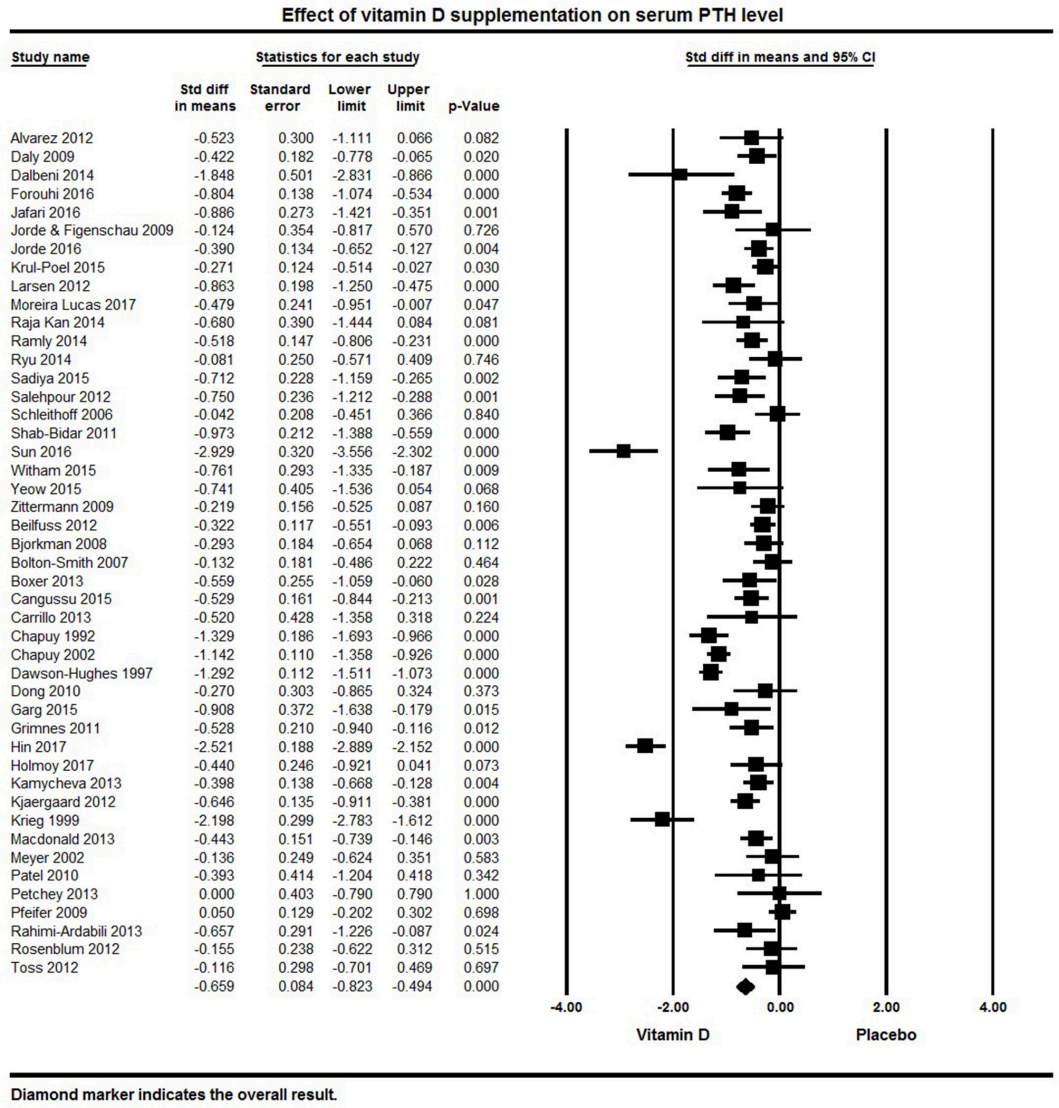
Forest plot detailing standardized mean difference for the impact of vitamin D on serum PTH level.

Forty-five papers reported serum PTH levels as a primary or secondary endpoint. The pooled effect size of vitamin D on serum PTH levels was −0.66 ± 0.08 ng/L (95% CI −0.82 to −0.50, *p* < 0.001, *I*^2^ = 87%) across all studies (Figure 8). Twenty eight individual studies reported a significant reduction in PTH levels with vitamin D supplementation (32, 50, 65, 68, 70, 73–76, 82, 84, 87, 89, 92, 96–100, 102, 105, 117, 120, 125, 127, 131, 133, 136), 15 reported a non-significant reduction in PTH (60, 66, 67, 77, 90, 95, 104, 109, 116, 118, 119, 122, 129, 135, 138) and two studies found no change or an increase in PTH levels (110, 139).

#### Vitamin D and hs-CRP

**Figure 9:**
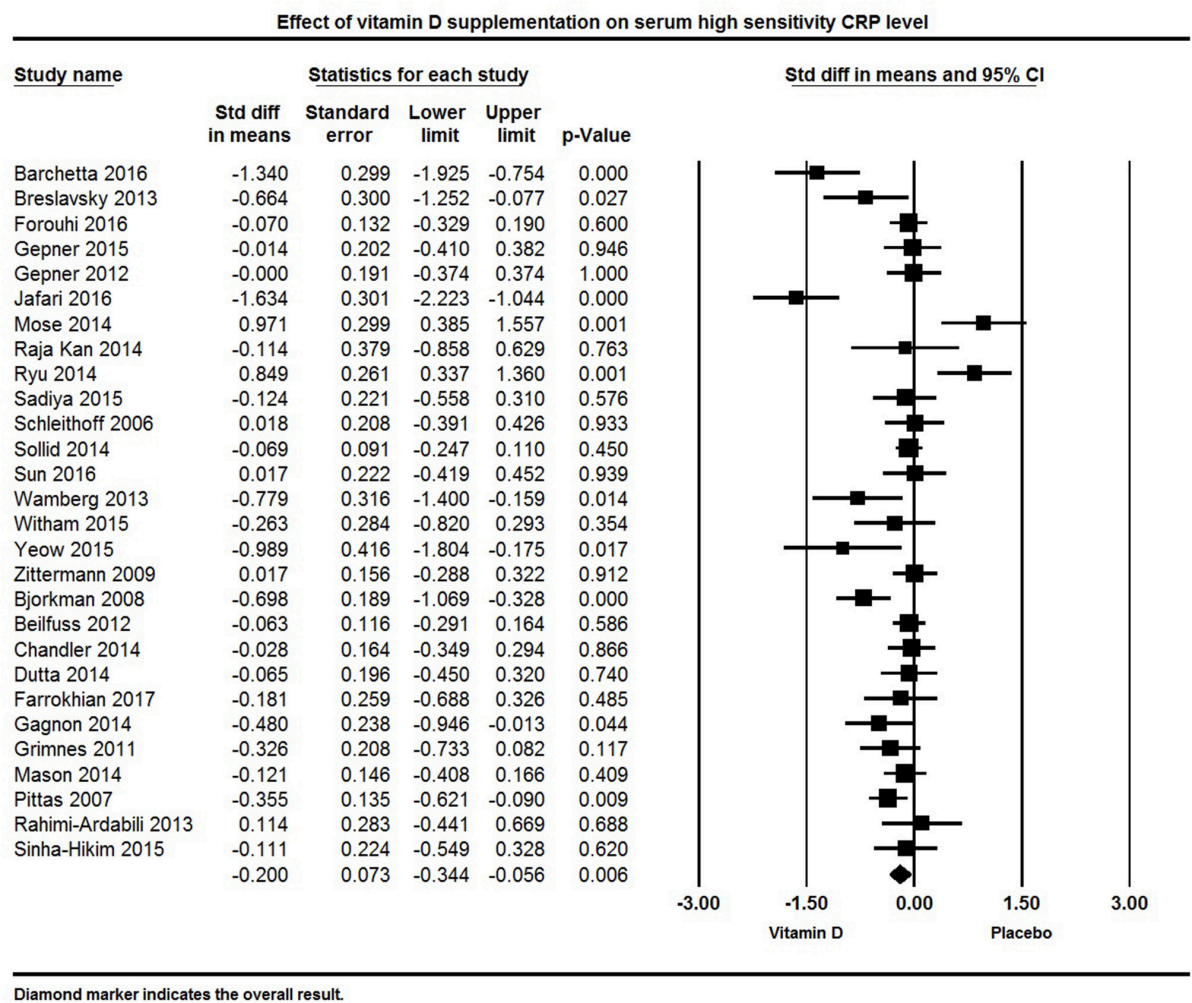
Forest plot detailing standardized mean difference for the impact of vitamin D on serum high sensitivity CRP level.

Twenty eight studies reported hs-CRP concentration as an outcome. The pooled effect size (standardized mean difference) of vitamin D supplementation on serum hs-CRP was −0.20 ± 0.07 mg/L (95% CI −0.34 to −0.06, *p* = 0.006, *I*^2^ = 73%) across all studies (Figure 9). Eight individual studies reported a significant reduction in serum hs-CRP following vitamin D supplementation (64, 66, 69, 83, 92, 112, 130, 133), 13 indicated a non-significant reduction in hs-CRP (65, 71, 72, 78, 80, 82, 86, 87, 116, 120, 126, 131, 137), and seven found either a null effect (85) or an increase in hs-CRP in the vitamin D supplemented group (106, 115, 119, 122, 127, 135).

#### Vitamin D and peak wave velocity

**Figure 10:**
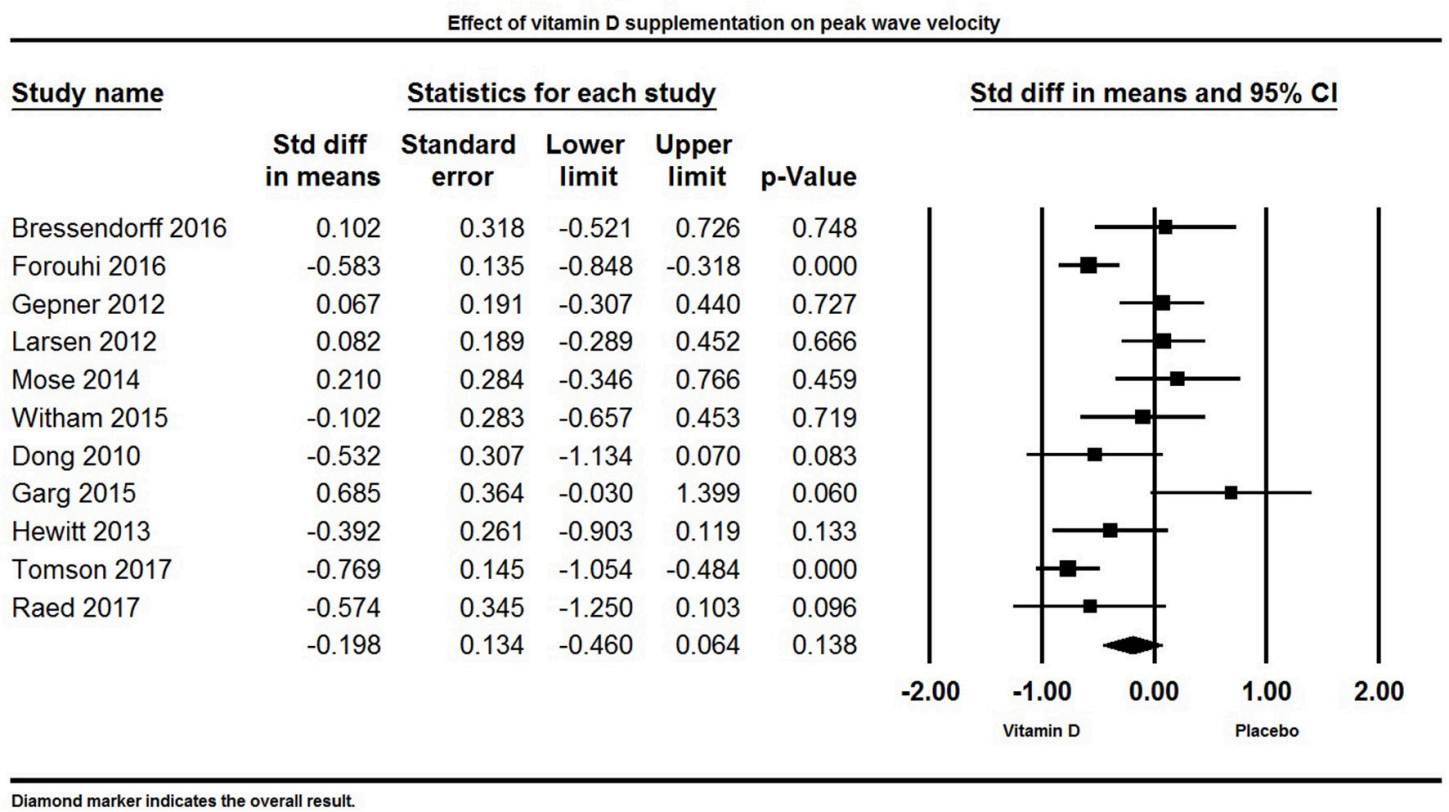
Forest plot detailing standardized mean difference for the impact of vitamin D on peak wave velocity.

Eleven papers reported PWV as a primary or secondary outcome. Overall, there was no significant effect of vitamin D supplementation on PWV. The pooled effect size of vitamin D on PWV was −0.20 ± 0.13 m/s [95% CI −0.46 to 0.06, *p* = 0.13, *I*^2^ = 72%) across all studies (Figure 10). Four individual studies reported a significant reduction in PWV in the group supplemented with vitamin D (77, 82, 114, 128), two studies found a non-significant trend for reduction in PWV (88, 131), and five trials did not find any significant effect of vitamin D on PWV (33, 84, 85, 100, 106).

#### Vitamin D and augmentation index

**Figure 11:**
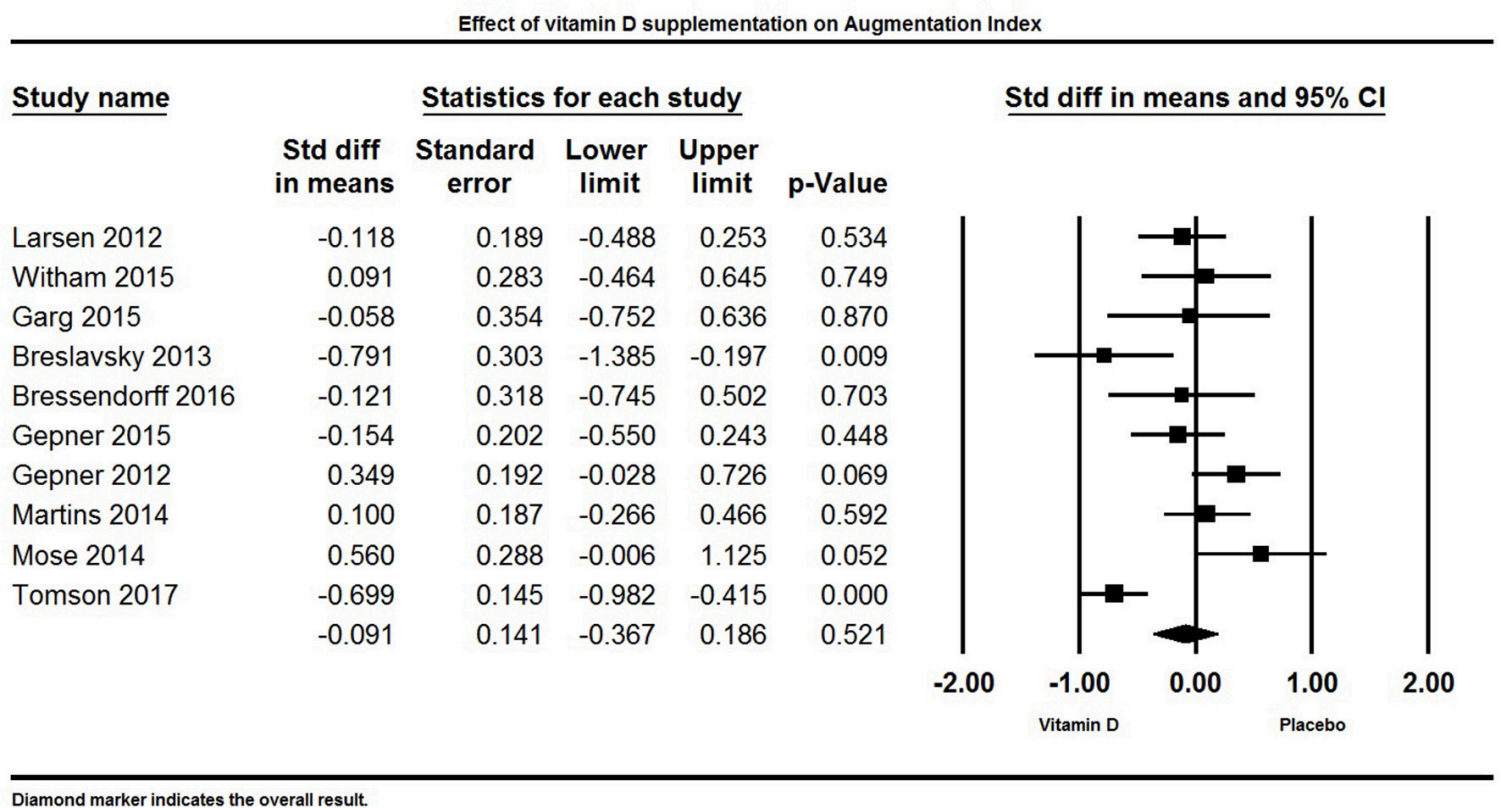
Forest plot detailing standardized mean difference for the impact of vitamin D on augmentation index.

Ten studies reported AI as an outcome (Figure 11). Overall, vitamin D supplementation did not have an effect on AI. The pooled estimate (standardized mean difference) of the effect of vitamin D administration on AI was −0.09 ± 0.14% (95% CI −0.37 to 0.20%, *p* = 0.52, *I*^2^ = 74%).

Two studies reported a significant decrease in AI (69, 128), two studies reported a significant increase in AI (85, 106), and six trials did not find any influence of vitamin D supplementation on AI (33, 84, 86, 100, 103, 131).

### Sensitivity analysis

Using leave-one-out sensitivity analysis, the effect size of vitamin D remained significant for blood pressure, lipid profile, serum PTH, and serum hs-CRP, confirming that difference between treatment groups is not due to the effect of any single study.

We then removed more than one study based on a number of possible outliers and repeated meta-analysis. For serum PTH, three studies were removed (89, 98, 127) and vitamin D supplementation still significantly lowered serum PTH (−0.53 ± 0.06 ng/L, 95% CI −0.65 to −0.41, *p* < 0.001). For serum hs-CRP, two studies were removed (64, 92) and we found significant reduction in serum hs-CRP following vitamin D supplementation (−0.12 ± 0.06 mg/L, 95% CI −0.24 to −0.003, *p* = 0.04). Three studies were removed as outliers for serum TC (32, 76, 134) and there was still a significant lowering effect of vitamin D supplementation on serum TC (−0.14 ± 0.04 mmol/L, 95% CI −0.22 to −0.06, *p* < 0.001). There was one outlier for serum LDL (92); after removing this study, serum LDL decreased following vitamin D supplementation by −0.08 ± 0.05 mmol/L (95% CI −0.18 to 0.01, *p* = 0.07). Salehpour (32) study was removed as an outlier for serum HDL and the overall vitamin D effect size was 0.06 ± 0.04 mmol/L (95% CI −0.01 to 0.12, *p* = 0.12). For systolic BP, three studies were removed (61, 76, 81) and there was a non-significant decreased trend in systolic BP following vitamin D supplementation (−0.04 ± 0.03 mmHg, 95% CI −0.09 to 0.02, *p* = 0.2). We did not identify any outliers for diastolic BP, PWV, AI, and serum TG.

### Publication bias

Visual inspection of funnel plot symmetry suggested potential publication bias for the comparison of systolic and diastolic BP, PTH, PWV, TC, HDL, and hs-CRP between vitamin D-administered and placebo groups, though funnel plots for AI, TG, and LDL looked symmetric (Figures S1–S10). Egger's linear regressions did not indicate publication bias. After adjusting the effect size using the Trim and Fill method for potential publication bias, except for PWV, the effect size of vitamin D on lowering CVD risk parameters increased (BP, hs-CRP, PTH, AI, TC, LDL) or remained unchanged (TG, HDL) (Table S2).

### Sub-group analysis

We investigated the effect of dose, achieved mean serum 25(OH)D, length of intervention, obesity, vitamin D deficiency, co-administration of calcium supplementation and age.

#### Achieved serum 25(OH)D concentrations at the end of the trial

We investigated the effect of achieved serum 25(OH)D as high vs. low based on median levels (Table 2). Serum 25(OH)D concentrations ≥86 nmol/L resulted in a significantly higher reduction in systolic (−0.15 ± 0.06 vs. −0.04 ± 0.04 mmHg, *p* = 0.05), diastolic BP (−0.12 ± 0.05 vs. −0.01 ± 0.04 mmHg, *p* = 0.04), PWV (−0.28 ± 0.16 vs. −0.004 ± 0.29 m/s, *p* = 0.08), and hs-CRP (−0.23 ± 0.09 vs. −0.11 ± 0.16 md/L, *p* = 0.07). AI, for which there was no significant effect overall, was found to be significantly lower among participants with serum 25(OH)D concentrations ≥86 nmol/L (−0.16 ± 0.2% vs. −0.005 ± 0.2%, *p* = 0.05). PTH and lipid changes did not significantly differ based on achieved serum 25(OH)D concentration.

**Table 2:** Meta-analysis and subgroup analysis of primary and secondary outcomes.

**Subgroup analysis**	**No. of study**	**No. of subjects**		**Standardized Mean difference (95% CI)**	***P value***	Between groups P value^*^
		**Vitamin D**	**Placebo**			
**SERUM 25(OH)D LEVEL AT FOLLOW-UP**						
**Systolic blood pressure**						
<86 nmol/L	17	1,008	993	−0.04 ± 0.04 (−0.12 to 0.05)	0.44	0.05^*^
≥86 nmol/L	22	1,390	1,414	−0.15 ± 0.06 (−0.29 to −0.01)	0.04	
**Diastolic blood pressure**						
<86 nmol/L	17	1,008	993	−0.01 ± 0.04 (−0.10 to 0.07)	0.75	0.04^*^
≥86 nmol/L	22	1,390	1,414	−0.12 ± 0.05 (−0.21 to −0.02)	0.01	
**Augmentation Index**						
<86 nmol/L	5	149	145	−0.005 ± 0.2 (−0.41 to 0.39)	0.98	0.05^*^
≥86 nmol/L	5	282	281	−0.16 ± 0.2 (−0.55 to 0.23)	0.42	
**Peak Wave Velocity**						
<86 nmol/L	4	179	181	−0.004 ± 0.29 (−0.56 to 0.56)	0.98	0.08
≥86 nmol/L	7	304	300	−0.28 ± 0.16 (−0.61 to 0.04)	0.08	
**Serum C-Reactive Protein**						
<86 nmol/L	8	355	352	−0.11 ± 0.16 (−0.42 to 0.21)	0.50	0.07
≥86 nmol/L	20	1,311	1,216	−0.23 ± 0.09 (−0.40 to −0.07)	0.006	
**Serum PTH**						
<86 nmol/L	25	1,387	1,388	−0.66 ± 0.11 (−0.88 to −0.44)	<0.001	0.36
≥86 nmol/L	21	1,429	1,332	−0.65 ± 0.13 (−0.90 to −0.40)	<0.001	
**Total Cholesterol (TC)**						
<86 nmol/L	23	981	971	−0.18 ± 0.09 (−0.35 to −0.01)	0.03	0.22
≥86 nmol/L	15	794	801	−0.10 ± 0.05 (−0.20 to −0.004)	0.04	
**Triglyceride (TG)**						
<86 nmol/L	21	854	847	−0.16 ± 0.08 (−0.32 to 0.008)	0.06	0.18
≥86 nmol/L	17	992	1,000	−0.08 ± 0.09 (−0.24 to 0.09)	0.38	
**HDL Cholesterol (HDL)**						
<86 nmol/L	21	943	936	0.10 ± 0.07 (−0.3 to 0.23)	0.13	0.45
≥86 nmol/L	18	1,024	1,032	0.07 ± 0.05 (−0.04 to 0.17)	0.24	
**LDL Cholesterol (LDL)**						
<86 nmol/L	20	821	809	−0.11 ± 0.06 (−0.22 to 0.002)	0.054	0.35
≥86 nmol/L	17	1,002	1,011	−0.09 ± 0.08 (−0.25 to 0.08)	0.29	
**VITAMIN D SUPPLEMENTATION DOSE**						
**Systolic blood pressure**						
<4,000 IU/d	25	1,672	1,664	−0.01 ± 0.03 (−0.08 to 0.06)	0.77	0.001^*^
≥4,000 IU/d	14	748	756	−0.31 ± 0.12 (−0.55 to −0.07)	0.01	
**Diastolic blood pressure**						
<4,000 IU/d	25	1,672	1,664	−0.03 ± 0.03 (−0.10 to 0.04)	0.43	0.05^*^
≥4,000 IU/d	14	748	756	−0.17 ± 0.09 (−0.35 to 0.01)	0.06	
**Augmentation index**						
<4,000 IU/d	8	315	307	0.007 ± 0.12 (−0.23 to 0.25)	0.95	0.07
≥4,000 IU/d	2	116	119	−0.46 ± 0.31 (−1.07 to 0.15)	0.13	
**Peak Wave Velocity**						
<4,000 IU/d	7	319	315	−0.13 ± 0.14 (−0.41 to 0.15)	0.38	0.29
≥4,000 IU/d	4	164	166	−0.32 ± 0.29 (−0.88 to 0.24)	0.27	
**Serum C-Reactive Protein**						
<4,000 IU/d	20	1,376	1,282	−0.16 ± 0.1 (−0.34 to 0.02)	0.07	0.05^*^
≥4,000 IU/d	8	290	286	−0.28 ± 0.1 (−0.48 to −0.08)	0.006	
**Serum PTH**						
<4,000 IU/d	31	2,134	2,041	−0.61 ± 0.1 (−0.80 to −0.42)	<0.001	0.21
≥4,000 IU/d	15	682	679	−0.77 ± 0.17 (−1.11 to −0.43)	<0.001	
**Total Cholesterol (TC)**						
<4,000 IU/d	24	1,318	1,308	−0.13 ± 0.05 (−0.24 to −0.03)	0.01	0.43
≥4,000 IU/d	14	457	464	−0.16 ± 0.13 (−0.42 to 0.11)	0.25	
**Triglyceride (TG)**						
<4,000 IU/d	25	1,402	1,393	−0.14 ± 0.07 (−0.29 to 0.003)	0.054	0.28
≥4,000 IU/d	13	444	454	−0.06 ± 0.1 (−0.26 to 0.13)	0.51	
**HDL Cholesterol (HDL)**						
<4,000 IU/d	26	1,523	1,514	0.11 ± 0.06 (0.003 to 0.22)	0.04	0.13
≥4,000 IU/d	13	444	454	0.03 ± 0.07 (−0.10 to 0.16)	0.67	
**LDL Cholesterol (LDL)**						
<4,000 IU/d	25	1,401	1,387	−0.13 ± 0.07 (−0.25 to 0.003)	0.055	0.14
≥4,000 IU/d	12	422	433	−0.04 ± 0.07 (−0.18 to 0.09)	0.54	
**DURATION OF INTERVENTION**						
**Systolic blood pressure**						
<6 months	16	750	743	−0.22 ± 0.1 (−0.42 to −0.02)	0.03	0.04^*^
≥6 months	23	1,648	1,664	−0.02 ± 0.03 (−0.09 to 0.05)	0.58	
**Diastolic Blood Pressure**						
<6 months	16	750	743	−0.15 ± 0.07 (−0.29 to −0.01)	0.03	0.02^*^
≥6 months	23	1,648	1,664	−0.03 ± 0.03 (−0.10 to 0.04)	0.35	
**Augmentation Index**						
<6 months	4	192	185	0.08 ± 0.11 (−0.14 to 0.30)	0.46	0.11
≥6 months	6	239	241	−0.20 ± 0.21 (−0.61 to 0.21)	0.34	
**Peak Wave Velocity**						
<6 months	6	287	282	−0.23 ± 0.16 (−0.54 to 0.08)	0.15	0.25
≥6 months	5	196	199	−0.12 ± 0.26 (−0.63 to 0.38)	0.63	
**Serum C-Reactive Protein**						
<6 months	7	336	331	−0.32 ± 0.19 (−0.70 to 0.05)	0.09	0.24
≥6 months	21	1,330	1,237	−0.17 ± 0.08 (−0.32 to −0.01)	0.03	
**Serum PTH**						
<6 months	11	402	414	−0.70 ± 0.08 (−0.85 to −0.54)	<0.001	0.41
≥6 months	35	2,414	2,306	−0.66 ± 0.1 (−0.86 to −0.46)	<0.001	
**Total Cholesterol (TC)**						
<6 months	15	619	628	−0.25 ± 0.12 (−0.48 to −0.02)	0.03	0.07
≥6 months	23	1,156	1,144	−0.10 ± 0.06 (−0.20 to 0.01)	0.07	
**Triglyceride (TG)**						
<6 months	14	505	514	−0.31 ± 0.09 (−0.48 to −0.13)	0.001	0.006^*^
≥6 months	24	1,341	1,333	−0.02 ± 0.07 (−0.16 to 0.12)	0.82	
**HDL Cholesterol (HDL)**						
<6 months	15	619	628	0.19 ± 0.09 (0.008 to 0.37)	0.04	0.04^*^
≥6 months	24	1,348	1,340	0.03 ± 0.04 (−0.05 to 0.11)	0.44	
**LDL Cholesterol (LDL)**						
<6 months	13	472	476	−0.14 ± 0.09 (−0.32 to 0.03)	0.11	0.18
≥6 months	24	1,351	1,344	−0.08 ± 0.06 (−0.20 to 0.04)	0.18	
**OBESITY (BMI** ≥ **30 KG/M**^2^**)**						
**Systolic blood pressure**						
Obese	19	999	999	−0.19 ± 0.09 (−0.37 to −0.01)	0.03	0.02^*^
Non-obese	20	1,298	1,408	−0.05 ± 0.04 (−0.12 to 0.03)	0.20	
**Diastolic blood pressure**						
Obese	19	999	999	−0.12 ± 0.06 (−0.23 to −0.01)	0.04	0.08
Non-obese	20	1,298	1,408	−0.04 ± 0.04 (−0.12 to 0.04)	0.31	
**Serum C-Reactive Protein**						
Obese	11	635	535	−0.18 ± 0.12 (−0.43 to 0.06)	0.13	0.45
Non-obese	17	1,031	1,033	−0.21 ± 0.09 (−0.39 to −0.03)	0.02	
**Serum PTH**						
Obese	15	842	741	−0.47 ± 0.06 (−0.58 to −0.35)	<0.001	0.22
Non-obese	31	1,974	1,979	−0.72 ± 0.12 (−0.94 to −0.49)	<0.001	
**Total Cholesterol (TC)**						
Obese	19	781	792	−0.10 ± 0.08 (−0.26 to 0.05)	0.19	0.17
Non-obese	19	994	980	−0.19 ± 0.08 (−0.34 to −0.03)	0.02	
**Triglyceride (TG)**						
Obese	19	1,013	1,028	−0.06 ± 0.07 (−0.21 to 0.08)	0.41	0.10
Non-obese	19	833	819	−0.18 ± 0.09 (−0.36 to 0.006)	0.059	
**HDL Cholesterol (HDL)**						
Obese	18	771	786	0.14 ± 0.07 (−0.006 to 0.28)	0.06	0.23
Non-obese	21	1,196	1,182	0.04 ± 0.05 (−0.06 to 0.14)	0.43	
**LDL Cholesterol (LDL)**						
Obese	16	716	727	−0.04 ± 0.05 (−0.14 to 0.07)	0.47	0.20
Non-obese	21	1,107	1,093	−0.14 ± 0.07 (−0.29 to 0.00)	0.05	
**VITAMIN D DEFICIENCY AT BASELINE (**<**50 NMOL/L)**						
**Systolic blood pressure**						
<50 nmol/L	26	1,345	1,363	−0.12 ± 0.06 (−0.25 to 0.01)	0.06	0.16
≥50 nmol/L	13	1,044	1,044	−0.06 ± 0.06 (−0.18 to 0.05)	0.27	
**Diastolic blood pressure**						
<50 nmol/L	26	1,345	1,363	−0.06 ± 0.05 (−0.16 to 0.03)	0.21	0.18
≥50 nmol/L	13	1,044	1,044	−0.08 ± 0.04 (−0.17 to 0.006)	0.07	
**Augmentation Index**						
<50 nmol/L	5	149	145	−0.005 ± 0.2 (−0.41 to 0.39)	0.97	0.10
≥50 nmol/L	5	282	281	−0.16 ± 0.2 (−0.55 to 0.23)	0.42	
**Peak Wave Velocity**						
<50 nmol/L	8	272	267	−0.19 ± 0.15 (−0.49 to 0.11)	0.22	0.42
≥50 nmol/L	3	211	214	−0.22 ± 0.30 (−0.81 to 0.38)	0.47	
**Serum C-Reactive Protein**						
<50 nmol/L	18	738	747	−0.20 ± 0.11 (−0.42 to 0.02)	0.07	0.45
≥50 nmol/L	10	928	821	−0.20 ± 0.09 (−0.38 to −0.007)	0.04	
**Serum PTH**						
<50 nmol/L	29	1,464	1,471	−0.81 ± 0.12 (−1.05 to −0.58)	<0.001	0.01^*^
≥50 nmol/L	17	1,352	1,249	−0.43 ± 0.10 (−0.63 to −0.22)	<0.001	
**Total Cholesterol (TC)**						
<50 nmol/L	28	1,069	1,071	−0.12 ± 0.07 (−0.26 to 0.02)	0.09	0.31
≥50 nmol/L	10	706	701	−0.20 ± 0.09 (−0.37 to −0.03)	0.02	
**Triglyceride (TG)**						
<50 nmol/L	28	1,140	1,146	−0.13 ± 0.07 (−0.26 to 0.006)	0.06	0.46
≥50 nmol/L	10	706	701	−0.10 ± 0.12 (−0.34 to 0.14)	0.41	
**HDL Cholesterol (HDL)**						
<50 nmol/L	29	1,261	1,267	0.05 ± 0.05 (−0.04 to 0.15)	0.31	0.09
≥50 nmol/L	10	706	701	0.17 ± 0.08 (0.006 to 0.33)	0.04	
**LDL Cholesterol (LDL)**						
<50 nmol/L	28	1,150	1,157	−0.05 ± 0.05 (−0.14 to 0.05)	0.32	0.05^*^
≥ 50 nmol/L	9	673	663	−0.24 ± 0.11 (−0.46 to −0.02)	0.03	
**CALCIUM CO-SUPPLEMENTATION**						
**Systolic blood pressure**						
Yes	7	512	514	−0.19 ± 0.2 (−0.58 to 0.19)	0.33	0.07
No	32	1,886	1,893	−0.07 ± 0.03 (−0.13 to −0.004)	0.04	
**Diastolic blood pressure**						
Yes	7	512	514	−0.12 ± 0.12 (−0.35 to 0.12)	0.34	0.33
No	32	1,886	1,893	−0.06 ± 0.03 (−0.13 to 0.003)	0.06	
**Serum C-Reactive Protein**						
Yes	8	627	530	−0.12 ± 0.13 (−0.38 to 0.13)	0.33	0.19
No	20	1,039	1,038	−0.24 ± 0.09 (−0.42 to −0.06)	0.01	
**Serum PTH**						
Yes	17	1,369	1,278	−0.58 ± 0.14 (−0.85 to −0.31)	<0.001	0.21
No	29	1,447	1,442	−0.71 ± 0.11 (−0.93 to −0.50)	<0.001	
**Total Cholesterol (TC)**						
Yes	8	383	392	−0.16 ± 0.10 (−0.37 to 0.04)	0.13	0.41
No	30	1,392	1,380	−0.14 ± 0.07 (−0.27 to −0.01)	0.03	
**Triglyceride (TG)**						
Yes	9	436	438	−0.18 ± 0.13 (−0.43 to 0.07)	0.16	0.11
No	29	1,410	1,409	−0.10 ± 0.07 (−0.23 to 0.03)	0.14	
**HDL Cholesterol (HDL)**						
Yes	10	468	470	0.07 ± 0.09 (−0.11 to 0.26)	0.42	0.39
No	29	1,499	1,498	0.09 ± 0.05 (−0.009 to 0.18)	0.07	
**LDL Cholesterol (LDL)**						
Yes	9	435	432	−0.11 ± 0.1 (−0.31 to 0.09)	0.28	0.38
No	28	1,388	1,388	−0.10 ± 0.06 (−0.21 to 0.02)	0.09	
**PARTICIPANT'S AGE (55 YEARS)**						
**Systolic blood pressure**						
<55 years	21	1,264	1,270	−0.14 ± 0.07 (−0.29 to −0.003)	0.04	0.07
≥55 years	18	1,134	1,137	−0.05 ± 0.05 (−0.15 to 0.05)	0.33	
**Diastolic blood pressure**						
<55 years	21	1,264	1,270	−0.08 ± 0.06 (−0.19 to 0.04)	0.17	0.21
≥55 years	18	1,134	1,137	−0.07 ± 0.04 (−0.15 to 0.01)	0.08	
**Augmentation Index**						
<55 years	4	122	115	0.04 ± 0.13 (−0.22 to 0.29)	0.76	0.31
≥55 years	6	309	311	−0.15 ± 0.21 (−0.56 to 0.27)	0.48	
**Peak Wave Velocity**						
<55 years	6	217	212	−0.21 ± 0.19 (−0.59 to 0.16)	0.27	0.45
≥55 years	5	266	269	−0.18 ± 0.21 (−0.59 to 0.24)	0.40	
**Serum C-Reactive Protein**						
<55 years	14	795	587	−0.14 ± 0.06 (−0.25 to −0.02)	0.01	0.48
≥55 years	14	871	881	−0.22 ± 0.13 (−0.48 to 0.04)	0.09	
**Serum PTH**						
<55 years	20	1,063	966	−0.65 ± 0.1 (−0.85 to −0.46)	<0.001	0.45
≥55 years	26	1,753	1,754	−0.65 ± 0.12 (−0.90 to −0.41)	<0.001	
**Total Cholesterol (TC)**						
<55 years	22	801	808	−0.17 ± 0.09 (−0.34 to 0.007)	0.06	0.34
≥55 years	16	974	964	−0.12 ± 0.07 (−0.25 to 0.008)	0.06	
**Triglyceride (TG)**						
<55 years	23	862	876	−0.14 ± 0.08 (−0.30 to 0.014)	0.07	0.37
≥55 years	15	984	971	−0.08 ± 0.09 (−0.26 to 0.09)	0.09	
**HDL Cholesterol (HDL)**						
<55 years	23	951	965	0.08 ± 0.06 (−0.04 to 0.19)	0.19	0.26
≥55 years	16	1,016	1,003	0.10 ± 0.06 (−0.03 to 0.22)	0.14	
**LDL Cholesterol (LDL)**						
<55 years	21	807	817	−0.04 ± 0.06 (−0.16 to 0.07)	0.47	0.10
≥55 years	16	1,016	1,003	−0.16 ± 0.08 (−0.31 to −0.008)	0.03	

*P-value represents within group comparison, subgroup analysis was not done for 2HPG and obesity (one study in obese subgroup), Independent T-test for between groups comparison*,

* *p-values adjusted by Bonferroni correction*.

#### Vitamin D supplementation dose

We investigated dose effect on outcomes by comparing trials in which subjects received ≥4,000 IU/d of vitamin D to those in which subjects received <4,000 IU/d (Table 2). Trials with vitamin D doses ≥4,000 IU/d had significantly greater reduction in systolic (−0.31 ± 0.12 vs. −0.01 ± 0.03 mmHg, *p* = 0.001), diastolic BP (−0.17 ± 0.09 vs. −0.03 ± 0.03 mmHg, *p* = 0.05), and hs-CRP (−0.28 ± 0.1 vs. −0.16 ± 0.1 mg/L, *p* = 0.05). AI was significantly lowered in trials with vitamin D doses ≥4,000 IU/d (−0.46 ± 0.31% vs. 0.007 ± 0.12%, *p* = 0.07).

#### Duration of intervention

To investigate whether the length of the trial influenced the effect of vitamin D supplementation on outcomes we investigated trials that supplemented for ≥6 months in comparison with those <6 months in duration (Table 2). In trials that assessed vitamin D supplementation for <6 months there was a significantly greater decrease in systolic (−0.22 ± 0.1 vs. −0.02 ±0.03 mmHg, *p* = 0.04), diastolic BP (−0.15 ± 0.07 vs. −0.03 ± 0.03 mmHg, *p* = 0.02), and TG (−0.31 ± 0.09 vs. −0.02 ± 0.07 mmol/L), and a greater increase in HDL (0.19 ± 0.09 vs. 0.03 ± 0.04 mmol/L, *p* = 0.04).

#### Obesity

We investigated whether outcomes differed between trials that enrolled an obese (BMI ≥ 30 kg/m^2^) population vs. non-obese (Table 2). In trials with obese subjects there was a significantly greater reduction in systolic (−0.19 ± 0.09 vs. −0.05 ± 0.04 mmHg, *p* = 0.02) and diastolic BP (−0.12 ± 0.06 vs. −0.04 ± 0.04 mmHg, *p* = 0.08). There was no significant difference in other outcomes based on obesity.

#### Vitamin D deficiency at baseline

We investigated whether the effect of vitamin D on outcomes was dependent on vitamin D deficiency at baseline by comparing trials vitamin D deficient subjects at baseline [serum 25(OH)D <50 nmol/L] vs. vitamin D sufficient subjects (Table 2). Vitamin D supplementation in trials with participants who were vitamin D deficient had a significantly greater reduction in PTH (−0.81 ± 0.12 vs. −0.43 ± 0.1 ng/L, *p* = 0.01), LDL (−0.24 ± 0.11 vs. −0.05 ± 0.05 mmol/L, *p* = 0.05) and AI (−0.16 ± 0.2% vs. −0.005 ± 0.2%, *p* = 0.10), and a greater increase in HDL (0.17 ± 0.08 vs. 0.05 ± 0.05 mmol/L, *p* = 0.09) in comparison with vitamin D sufficient participants.

#### Calcium co-administration

We investigated whether calcium co-administration influenced outcomes by comparing those trials with those that supplemented with vitamin D alone (Table 2). Participants supplemented with both vitamin D and calcium had a significantly greater reduction in systolic BP (−0.19 ± 0.2 vs. −0.07 ±0.03 mmHg, *p* = 0.07) and TG levels (−0.18 ± 0.13 vs. −0.10 ±0.07 mmol/L, *p* = 0.11), compared with those who received vitamin D alone. There was no difference among the remaining parameters.

#### Effect of participants' age

Age itself is a risk factor for CVD and thus we compared trials that enrolled populations ≥55 y vs. <55 y. Vitamin D supplementation in trials with populations <55 y had significantly greater reduction in systolic BP (−0.14 ± 0.07 vs.−0.05 ± 0.05 mmHg, *p* = 0.07). There was no significant difference in other outcomes based on participant age grouping.

## Discussion

As the leading cause of death and disability worldwide, cardiovascular disease (CVD) is a major public health burden (140). Much effort has been devoted to identifying modifiable risk factors to prevent CVD. Vitamins may have a role in the prevention and treatment of CVD. Antioxidant vitamins such as vitamin C, vitamin E, folic acid and vitamin B6 might decrease the rate of oxidative stress, a key component of atherosclerosis and CVD (14). Vitamin D and folic acid can inhibit inflammation with their anti-atherogenic effects. Vitamin E can inhibit platelet aggregation and B vitamins might have anti-thrombotic activity by lowering serum homocysteine levels (14, 141, 142). Among these, vitamin D, with its deficiency highly prevalent worldwide and having many pleiotropic effects, has been associated with CVD prevention in different community settings. Vitamin D deficiency impairs vascular function and is strongly associated with the heightened risks of various cardiovascular diseases such as hypertension, metabolic syndrome, heart failure, and stroke (3, 24).

Evidence suggests that vitamin D exerts beneficial cardiovascular effects through many pathways. Improved vitamin D status reduces RAAS activity and lowers blood pressure, it has anti-inflammatory, anti-proliferative, anti-hypertrophic, anti-fibrotic and anti-thrombotic impacts as well (111). Following vitamin D supplementation, suppression of renin production and downregulation of RAAS directly impacts myocardium and vasculature through modulating hypertrophic stimuli (143). Vitamin D inhibits the proliferation of vascular smooth muscle cells through influx of calcium into the cells, thus preserving endothelial function (144). Antihypertensive benefits of vitamin D include suppression of RAAS, an anti-proteinuric effect, a direct effect on endothelial cells and calcium metabolism as well as preventing secondary hyperparathyroidism (145, 146). Vitamin D may have both direct and indirect impacts on modifying lipid profiles. Vitamin D supplementation might decrease serum levels of triglyceride via increasing the activity of lipoprotein lipase in adipose tissue (147). Also, through improving calcium absorption, vitamin D might reduce fatty acid absorption via the formation of insoluble calcium-fatty acid complexes in the gut leading to decreased LDL cholesterol levels (148). Yet, despite these observations, evidence linking corrections to vitamin D status with improved cardiometabolic parameters is somewhat inconclusive (24, 43, 149).

Considering the alternate postulation, vitamin D deficiency might be a consequence of chronic conditions such as inflammation. There is a bacterial pathogenesis theory explains that intracellular bacteria commonly seen in chronic inflammation might invade different nucleated cells and affect vitamin D metabolism and its endocrine function resulting in low vitamin D status. This occurs concurrent to increased production of 1,25(OH)2D which is required for upregulating vitamin D receptors to transcribes more adenosine monophosphate. And more 25(OH)D should be metabolized in this process leading to low vitamin D status (150, 151). In another study conducted on the patients recovering from knee arthroplasty, there was a significant reduction in serum 25(OH)D levels during the process of systemic inflammatory response in these patients after surgery (152). Sattar et al. (153) also mentioned that vitamin D is an acute phase reactant and declines with the increase in inflammatory cytokine in different chronic conditions. Several mechanisms including decreased vitamin D carrier proteins, increased conversion of 25(OH)D to 1,25(OH)2D and hemodilution could be responsible for this reduction (154, 155). However for CVD, using Hill's criteria for causality, Weyland found that all relevant Hill criteria are satisfied suggesting low 25(OH)D level is an independent risk factor for CVD (156).

To better understand this incongruence, we analyzed 81 studies that evaluated the effect of vitamin D supplementation on various cardiometabolic risk parameters, including blood pressure, serum PTH, hs-CRP, lipid profile, and arterial PWV and AI. Unlike many previous studies, we imposed several strict inclusion criteria to select only well-designed trials. Overall, vitamin D supplementation was found to improve cardiovascular risk factors. Specifically, vitamin D supplementation, with doses above 4,000 IU/d and increased serum 25(OH)D concentrations ≥86 nmol/L decreased systolic and diastolic blood pressure, serum PTH, serum hs-CRP and improved lipid profiles (total cholesterol, triglyceride, HDL and LDL). Markers of arterial stiffness (PWV and AI) may also improve with vitamin D supplementation.

Subgroup analyses revealed that the co-administration of calcium with vitamin D led to greater reductions in blood pressure. The combination of calcium with vitamin D has been suggested to improve blood pressure by facilitating calcium absorption into the blood stream and optimizing serum calcium and PTH levels (157, 158). Although the greatest benefits of vitamin D supplementation can be achieved in vitamin D deficient populations, such that we observed as the lowering impact of vitamin D on serum PTH. Remarkably, we also found notable improvements in lipid profile in participants considered vitamin D sufficient prior to intervention. Further, individuals who were obese at baseline had a greater reduction in blood pressure, likely due to the higher percentage of obese individuals that are pre-hypertensive or hypertensive (159) and the higher daily doses of vitamin D provided to obese participants (61, 76, 81).

The results of our study compare closely with those from a number of recent meta-analyses. Jafari et al. (93), for instance, found significant reductions in the serum total cholesterol, triglyceride, and LDL levels of type 2 diabetics following vitamin D supplementation. Studying heterogeneous populations that consisted of healthy individuals, pregnant women, bedridden elderly people and those with different diseases (e.g., diabetes, heart failure, PCOS, and insulin resistant condition), both Chen et al. (160) and Rodriguez et al. (161) found that vitamin D supplementation significantly decreased inflammatory markers (i.e., hs-CRP). Chen et al. (160) additionally concluded that vitamin D supplementation led to a significantly greater reduction among those with baseline hs-CRP levels ≥5 mg/l. This is in line with increased hs-CRP levels in diabetic patients (64, 69, 92). Diabetes usually results in higher levels of hs-CRP and lower levels of 25(OH)D concentrations suggesting a larger effect size in subjects with this condition. Moreover, Wu et al. (162) and Witham et al. (163) found a significant modest reduction in blood pressure following vitamin D supplementation. Vitamin D supplementation may affect arterial stiffness and vascular aging through decreased synthesis of angiotensin II, following inhibition of RAAS, to increase vascular tone and arterial stiffness (164). However, limited data to assess the impact of vitamin D supplementation on the markers of arterial stiffness (PWV and AI) were inconclusive (43, 165), and may be due to inappropriate study design including insufficient duration of supplementation and insufficient power (119).

In contrast, Beveridge et al. (21) found no significant reduction in blood pressure after participants whose mean SBP was ≥140 mm Hg at baseline were supplemented with vitamin D. Of note, there are significant methodological differences in our approach. Beveridge et al. (21) included trials that combined vitamin D with antihypertensive drugs, administered large bolus doses to elderly populations, included subjects with resistant HTN, and/or supplemented with very low doses of vitamin D (i.e., 600 IU). These issues could mask any effects of vitamin D supplementation or simply not lead to any observable benefits. Our strict inclusion criteria resulted in the exclusion of 14 of the 27 studies that were analyzed by Beveridge et al. We had similar concerns with the meta-analysis conducted by Wang et al. (26) who reported that vitamin D supplementation led to a statistically significant increase in LDL and included RCTs that provided very low doses of vitamin D (i.e., 300 IU) or supplemented for durations considered too short (i.e., 42 days). Of the 12 studies included, and as mentioned by the authors, none were sufficiently powered to detect changes in CVD outcomes. The current meta-analysis revealed significant impact of vitamin D supplementation on lipid profiles with increased HDL and reduced LDL and TG.

Many observational studies support an association between cardiovascular risk factors and low vitamin D status. Perhaps most importantly, what constitutes vitamin D deficiency and repletion is somewhat debateable and, at times, contentious. The Institute of Medicine issues dietary recommendations, such as the Recommended Dietary Allowance (RDA), at the request of the U.S. and Canadian governments. In 2010, the Institute of Medicine set the RDA for vitamin D at 600 IU per day for individuals between the age of 1 and 70 (48). This RDA is assumed to achieve serum 25(OH)D levels of ≥50 nmol/l in 97.5% of the population. The methodology used to calculate this RDA, however, has been deemed erroneous (166) and estimates of much higher magnitude have been calculated by others – 3,875 IU/day (167) to 8,895 IU/day (168).

Similarly, the definition of what is an “optimal” serum 25(OH)D concentration is also controversial. Serum 25(OH)D concentrations >75 nmol/l (12) and >80 nmol/l (28) have been suggested as necessary for lipid and cardiovascular health. Serum 25(OH)D concentrations of 100–150 are defined as optimal by the U.S. Endocrine Society with values below 75 nmol/l deemed insufficient (169, 170). The results of the present meta-analysis suggest that serum 25(OH)D concentrations ≥86 nmol/L are optimal for reductions in blood pressure, markers of arterial stiffness, and reductions in hs-CRP. It is important to note that these serum 25(OH)D concentrations were achieved with vitamin D supplement doses ≥4,000 IU/d—the current tolerable upper level of intake. Using the standards of the U.S. Endocrine Society, 27 of the 81 included studies in our meta-analyses reached optimal 25(OH)D levels post-supplementation, and only 16 had post-supplement 25(OH)D levels that were insufficient.

The duration of supplementation is an important factor in assessments of vitamin D. With a half-life of 2 months, to achieve and maintain a steady serum 25(OH)D concentration requires a follow-up period of at least 3 months. Here, we included trials that ranged from 3 months to 5 years of intervention in the meta-analysis. Somewhat surprisingly, we found better improvement in some outcomes (blood pressure and lipids) in trials that were less than 6 months, although this is likely related to higher compliance in short-term interventions (171). Improvements in blood pressure and lipid profile were also witnessed in a short, 3 month vitamin D intervention in obese PCOS patients (116). The women received 12,000 IU/d of vitamin D for an increase in their serum 25(OH)D of 50–168 nmol/L. In contrast, a 5 year trial of obese and vitamin D-insufficient prediabetics provided 2,800 IU/d of vitamin D found no change in blood pressure (50). It is known that overweight and obese individuals require two to three times the amount of vitamin D to increase serum 25(OH)D concentrations to the same extent as those with a normal BMI (31, 47).

The present study has several strengths and limitations. Even after enforcing a strict inclusion criteria, the included studies varied with regard to participant age, serum 25(OH)D concentrations at baseline, concurrent use of other nutrients or medication, and overall health status. We used a random-effect model and performed sensitivity analyses to mitigate these limitations. For some of the studies, cardiovascular outcomes of interest were secondary outcomes or the trial was not of sufficient power to detect a change in these outcomes. Many of the studies also did not describe dietary intakes, season of treatment, or sun exposure. Further, some included trials assessed relatively small populations (10–13 participants per intervention group), but taken together offer support to the larger trials. Strengths include the large sample and the consideration of a wide variety of CVD risk parameters from at least 28 clinical trials for each CVD outcome (with the exception of arterial PWV and AI).

## Conclusion

Vitamin D deficiency is a highly prevalent condition and is independently associated with most CVD risk factors. The present meta-analysis demonstrated that vitamin D supplementation improved serum 25(OH)D concentrations significantly lowered blood pressure, serum PTH, hs-CRP, TC, LDL, and TG and increased HDL. Vitamin D supplementation also appears to improve arterial stiffness (PWV), but large and well-designed RCTs are required to confirm these findings. The present analysis suggests that for improvements in CVD risk factors vitamin D supplementation ≥4,000 IU/d and achieved serum 25(OH)D concentrations ≥86 nmol/L are required.

## Author contributions

NM, JR, and SK designed the study, NM and JR searched databases and performed the selection of studies. NM, JR, and SK wrote the manuscript. NM analyzed the data. SK and JR critically evaluated the review, commented on it, and approved the last version. All authors reviewed and approved the final manuscript. SK is the guarantor of this study.

### Conflict of interest statement

The authors declare that the research was conducted in the absence of any commercial or financial relationships that could be construed as a potential conflict of interest.
