# Supplementary material for: Vitamin D Supplementation, Serum 25(OH)D Concentrations and Cardiovascular Disease Risk Factors: A Systematic Review and Meta-Analysis
Source: Front Cardiovasc Med. 2018 Jul 12;5:87. doi: 10.3389/fcvm.2018.00087 (PMC6052909; doi:10.3389/fcvm.2018.00087)
Supplement: Supplementary file 1 [file Table_1.DOCX]

**Table S1.** Evaluation of the risk of bias for each included study (Cochrane checklist)

| **Study** | **Random sequence generation** | **Allocation concealment** | **Blinding of participants/ personnel** | **Blinding of outcome assessment** | **Incomplete outcome addressed** | **Comparability of treatment groups** | **Inclusion, exclusion criteria** | **Selective reporting** | **Intention- To- Treat** |
| --- | --- | --- | --- | --- | --- | --- | --- | --- | --- |
| Alvarez 2012 | + | + | + | + | + | + | + | - | + |
| Al-Sofiani 2015 | + | + | + | + | + | + | + | - | ? |
| Al-Zahrani 2014 | + | + | + | + | + | + | + | - | ? |
| Arora 2015 | + | + | + | + | + | + | + | ? | + |
| Barchetta 2016 | + | + | + | + | + | + | + | - | ? |
| Beilfuss 2012 | + | + | ? | ? | + | + | + | - | ? |
| Bjorkman 2008 | + | + | + | + | + | + | + | - | ? |
| Bolton-Smith 2007 | + | + | + | + | + | + | + | - | ? |
| Boxer 2013 | + | + | + | + | + | + | + | - | ? |
| Breslavsky 2013 | + | + | + | + | + | + | + | - | ? |
| Bressendorff 2016 | + | + | + | + | + | + | + | - | ? |
| Cangussu 2015 | + | + | + | + | + | + | + | - | + |
| Carrillo 2013 | + | + | + | + | + | + | + | - | ? |
| Chandler 2014 | + | + | + | + | + | + | + | - | ? |
| Chapuy 1992 | + | + | ? | ? | + | + | + | - | + |
| Chapuy 2002 | + | + | + | + | + | + | + | - | + |
| Daly 2009 | + | ? | ? | + | + | + | + | - | + |
| Dalbeni 2014 | + | + | + | + | + | + | + | - | ? |
| Dawson-Hughes 1997 | + | + | + | ? | + | + | + | - | + |
| Dong 2010 | + | + | +/- | + | + | + | + | - | + |
| Dutta 2014 | + | + | - | ? | - | + | + | - | ? |
| El-Hajj 2016 | + | + | + | + | + | + | + | - | + |
| Farrokhian 2017 | + | + | + | + | + | + | + | - | + |
| Forman 2013 | + | + | + | ? | + | + | + | - | + |
| Forouhi 2016 | + | + | + | + | + | + | + | - | + |
| Gagnon 2014 | + | + | + | + | + | + | + | - | - |
| Garg 2015 | + | + | + | ? | + | + | + | - | ? |
| Gepner 2012 | + | + | + | + | + | + | + | - | + |
| Gepner 2015 | + | + | + | + | ? | + | + | - | + |
| Grimnes 2011 | + | + | + | + | + | + | + | - | - |
| Hewitt 2013 | + | + | + | + | + | + | + | - | + |
| Hin 2017 | + | + | + | + | + | + | + | - | + |
| Holmoy 2017 | + | + | + | ? | ? | + | + | - | ? |
| Islam 2014 | + | + | + | + | + | + | + | - | ? |
| Jafari 2016 | + | + | + | + | + | + | + | - | ? |
| Jamilian 2017 | + | + | + | + | ? | + | + | - | - |
| Jorde & Figenschau 2009 | + | + | ? | ? | + | + | + | - | ? |
| Jorde 2016 | + | + | ? | ? | + | + | + | - | + |
| Kamycheva 2013 | + | + | ? | ? | + | + | + | - | ? |
| Kjaergaard 2012 | + | + | ? | ? | + | + | + | - | + |
| Krieg 1999 | + | + | - | ? | + | + | + | - | - |
| Krul-Poel 2015 | + | + | + | + | + | + | + | - | + |
| Larsen 2012 | + | + | + | + | + | + | + | - | ? |
| Lorvand Amiri 2016 | + | + | + | + | + | + | + | - | ? |
| Macdonald 2013 | + | + | + | + | + | + | + | - | + |
| Martins 2014 | + | + | + | + | + | + | + | - | + |
| Mason 2014 | + | + | + | - | + | + | + | - | + |
| Meyer 2002 | + | + | + | + | + | + | + | - | + |
| Moreira Lucas 2017 | + | + | + | ? | + | + | + | - | + |
| Mose 2014 | + | + | + | + | + | + | + | - | ? |
| Munoz- Aguirre 2015 | + | + | + | ? | + | + | + | - | + |
| Nikooyeh 2011 | + | + | + | + | + | + | + | - | - |
| Patel 2010 | + | + | ? | ? | + | + | + | - | ? |
| Petchey 2013 | + | + | + | + | + | + | + | - | ? |
| Pfeiffer 2009 | + | + | + | + | ? | + | + | - | + |
| Pittas 2007 | + | + | + | + | + | + | + | - | ? |
| Qin 2015 | + | + | + | + | + | + | + | - | + |
| Raed 2017 | + | + | + | + | + | + | + | - | + |
| Rahimi-Ardabili 2013 | + | + | + | + | + | + | + | - | ? |
| Raja Khan 2014 | + | + | + | + | + | + | + | - | + |
| Ramly 2014 | + | + | + | + | + | + | + | - | + |
| Rosenblum 2012 | + | + | + | + | + | + | + | - | + |
| Ryu 2014 | + | + | + | + | + | + | + | - | ? |
| Sadiya 2015 | + | + | + | + | + | + | + | - | - |
| Salekzamani 2016 | + | + | + | + | + | + | + | - | ? |
| Salehpour 2012 | + | + | + | + | + | + | + | - | ? |
| Schleitoff 2006 | + | + | + | + | + | + | + | - | ? |
| Scragg 2014 | + | + | + | + | + | + | + | - | ? |
| Seibert 2017 | + | + | + | + | + | + | + | - | ? |
| Shab-Bidar 2011 | + | + | + | + | ? | + | + | - | - |
| Sinha-Hikim 2015 | + | + | + | + | ? | + | + | - | ? |
| Sollid 2014 | + | + | + | + | + | + | + | - | + |
| Sun 2016 | + | + | + | + | + | + | + | - | ? |
| Tomson 2017 | + | + | + | + | + | + | + | - | + |
| Toss 2012 | + | + | + | + | + | + | + | - | ? |
| Wamberg 2013 | + | + | + | ? | + | + | + | - | + |
| Witham 2015 | + | + | + | + | + | + | + | - | + |
| Wood 2012 | + | + | + | + | + | + | + | - | + |
| Yeow 2015 | + | + | + | + | + | + | + | - | + |
| Yousefi Rad 2014 | + | + | + | + | ? | + | + | - | + |
| Zitterman 2009 | + | + | + | + | + | + | + | - | ? |
