# Supplementary material for: Vitamin D Supplementation, Serum 25(OH)D Concentrations and Cardiovascular Disease Risk Factors: A Systematic Review and Meta-Analysis
Source: Front Cardiovasc Med. 2018 Jul 12;5:87. doi: 10.3389/fcvm.2018.00087 (PMC6052909; doi:10.3389/fcvm.2018.00087)
Supplement: Supplementary file 2 [file Table_2.DOCX]

**Table S2**. Egger’s test and effect size of vitamin D after Trim and Fill adjustment (sensitivity analysis)

| **Parameter** | **Egger’s test** | | | | | **Trim & Fill adjustment** | | |  |
| --- | --- | --- | --- | --- | --- | --- | --- | --- | --- |
|  | **Intercept** | **SE** | **95% CI** | **t** | **P value** | **Effect size** | **95% CI** | **P value** | **# studies imputed** |
| Systolic BP | -0.89 | 0.57 | -2.05 to 0.28 | -1.54 | 0.13 | -0.121 | -0.21 to -0.03 | 0.007 | 3 |
| Diastolic BP | -0.01 | 0.45 | -0.94 to 0.91 | -0.03 | 0.97 | -0.106 | -0.17 to -0.04 | 0.002 | 6 |
| hs-CRP | -1.29 | 0.99 | -3.33 to 0.74 | -1.31 | 0.20 | -0.291 | -0.45 to -0.13 | <0.001 | 3 |
| PTH | -1.32 | 1.22 | -3.79 to 1.14 | -1.08 | 0.29 | -0.970 | -1.16 to -0.78 | <0.001 | 13 |
| PWV | 3.04 | 1.49 | -0.34 to 6.42 | 2.04 | 0.07 | -0.198 | -0.46 to 0.06 | 0.14 | 0 |
| AI | 2.18 | 2.25 | -3.02 to 7.38 | 0.97 | 0.36 | -0.283 | -0.56 to -0.005 | 0.04 | 3 |
| TC | -0.26 | 0.70 | -1.68 to 1.16 | -0.37 | 0.71 | -0.205 | -0.32 to -0.09 | 0.001 | 4 |
| TG | -1.43 | 0.77 | -2.99 to 0.12 | -1.87 | 0.07 | -0.119 | -0.23 to -0.003 | 0.04 | 0 |
| HDL | 0.77 | 0.58 | -0.41 to 1.96 | 1.33 | 0.19 | 0.085 | 0.00 to 0.17 | 0.04 | 0 |
| LDL | 0.70 | 0.67 | -0.67 to 2.07 | 1.03 | 0.31 | -0.132 | -0.23 to -0.03 | 0.009 | 3 |

SE=standard error, hs-CRP=high sensitivity C-reactive protein, PTH=parathyroid hormone, PWV=peak wave velocity, AI=augmentation index, TC=total cholesterol, TG=triglyceride, HDL=HDL-cholesterol, LDL=LDL-cholesterol
