# Supplementary material for: Vitamin D Supplementation, Serum 25(OH)D Concentrations and Cardiovascular Disease Risk Factors: A Systematic Review and Meta-Analysis
Source: Front Cardiovasc Med. 2018 Jul 12;5:87. doi: 10.3389/fcvm.2018.00087 (PMC6052909; doi:10.3389/fcvm.2018.00087)
Supplement: Supplementary file 3 [file Data_Sheet_1.DOCX]

(a) (b)

**Figure S1**. Funnel plot detailing publication bias in the selected studies for systolic BP analysis. (a) Funnel plot of SE by standardized mean difference; closed circles represent observed published studies. (b) Trim-and-fill method to impute for potentially missing studies; open circles represent observed published studies.

(a) (b)

**Figure S2**. Funnel plot detailing publication bias in the selected studies for diastolic BP analysis. (a) Funnel plot of SE by standardized mean difference; closed circles represent observed published studies. (b) Trim-and-fill method to impute for potentially missing studies; open circles represent observed published studies.

(a) (b)

**Figure S3**. Funnel plot detailing publication bias in the selected studies for parathyroid hormone (PTH) analysis. (a) Funnel plot of SE by standardized mean difference; closed circles represent observed published studies. (b) Trim-and-fill method to impute for potentially missing studies; open circles represent observed published studies.

(a) (b)

**Figure S4**. Funnel plot detailing publication bias in the selected studies for Peak Wave Velocity (PWV) analysis. (a) Funnel plot of SE by standardized mean difference; closed circles represent observed published studies. (b) Trim-and-fill method to impute for potentially missing studies; open circles represent observed published studies.

(a) (b)

**Figure S5**. Funnel plot detailing publication bias in the selected studies for Augmentation Index (AI) analysis. (a) Funnel plot of SE by standardized mean difference; closed circles represent observed published studies. (b) Trim-and-fill method to impute for potentially missing studies; open circles represent observed published studies.

(a) (b)

**Figure S6**. Funnel plot detailing publication bias in the selected studies for hs-CRP analysis. (a) Funnel plot of SE by standardized mean difference; closed circles represent observed published studies. (b) Trim-and-fill method to impute for potentially missing studies; open circles represent observed published studies.

(a) (b)

**Figure S7**. Funnel plot detailing publication bias in the selected studies for total cholesterol (TC) analysis. (a) Funnel plot of SE by standardized mean difference; closed circles represent observed published studies. (b) Trim-and-fill method to impute for potentially missing studies; open circles represent observed published studies.

(a) (b)

**Figure S8**. Funnel plot detailing publication bias in the selected studies for triglyceride (TG) analysis. (a) Funnel plot of SE by standardized mean difference; closed circles represent observed published studies. (b) Trim-and-fill method to impute for potentially missing studies; open circles represent observed published studies.

(a) (b)

**Figure S9**. Funnel plot detailing publication bias in the selected studies for HDL-cholesterol (HDL) analysis. (a) Funnel plot of SE by standardized mean difference; closed circles represent observed published studies. (b) Trim-and-fill method to impute for potentially missing studies; open circles represent observed published studies.

(a) (b)

**Figure S10**. Funnel plot detailing publication bias in the selected studies for LDL-cholesterol (LDL) analysis. (a) Funnel plot of SE by standardized mean difference; closed circles represent observed published studies. (b) Trim-and-fill method to impute for potentially missing studies; open circles represent observed published studies.
